# Pre-metastatic cancer exosomes induce immune surveillance by patrolling monocytes at the metastatic niche

**DOI:** 10.1038/s41467-017-01433-3

**Published:** 2017-11-06

**Authors:** Michael P. Plebanek, Nicholas L. Angeloni, Elena Vinokour, Jia Li, Anna Henkin, Dalia Martinez-Marin, Stephanie Filleur, Reshma Bhowmick, Jack Henkin, Stephen D. Miller, Igal Ifergan, Yesung Lee, Iman Osman, C. Shad Thaxton, Olga V. Volpert

**Affiliations:** 1 Department of Urology, Northwestern University Feinberg School of Medicine, 303 E. Chicago Ave, Chicago, IL 60611 USA; 2 Simpson-Querrey Institute for Bionantechnology in Medicine, 303 E. Superior St, Chicago, IL 60611 USA; 3 The Department for Health and Technology, Institute for Medical Engineering and Science, Massachusetts Institute of Technology, 45 Carleton St., Cambridge, MA 02139 USA; 4 Department of Urology, Texas Tech University Health Sciences Center, 3601 4th St, Lubbock, TX 79430-6591 USA; 5 Department of Cancer Biology, University of Texas MD Anderson Cancer Center, 1881 East Rd, Houston, TX 77054 USA; 6 Chemistry of Life Processes Institute, Northwestern University, 2145 Sheridan Rd, Evanston, IL 60208 USA; 7 Department of Microbiology-Immunology, Northwestern University Feinberg School of Medicine, 303 E. Chicago Ave, Chicago, IL 60611 USA; 8 Department of Dermatology, Northwestern University Feinberg School of Medicine, 303 E. Chicago Ave, Chicago, IL 60611 USA; 9 The Ronald O. Perelman Department of Dermatology, New York University School of Medicine, 240 East 38th Street, New York, NY 10016 USA; 10 Laura and Isaac Perlmutter Cancer Center, New York University, Langone Medical Center, 160 East 34th Street, New York, NY 10016 USA; 11 Northwestern University International Institute for Nanotechnology, 2145 Sheridan Rd., Evanston, IL 60208 USA

## Abstract

Metastatic cancers produce exosomes that condition pre-metastatic niches in remote microenvironments to favor metastasis. In contrast, here we show that exosomes from poorly metastatic melanoma cells can potently inhibit metastasis to the lung. These “non-metastatic” exosomes stimulate an innate immune response through the expansion of Ly6C^low^ patrolling monocytes (PMo) in the bone marrow, which then cause cancer cell clearance at the pre-metastatic niche, via the recruitment of NK cells and TRAIL-dependent killing of melanoma cells by macrophages. These events require the induction of the Nr4a1 transcription factor and are dependent on pigment epithelium-derived factor (PEDF) on the outer surface of exosomes. Importantly, exosomes isolated from patients with non-metastatic primary melanomas have a similar ability to suppress lung metastasis. This study thus demonstrates that pre-metastatic tumors produce exosomes, which elicit a broad range of PMo-reliant innate immune responses via trigger(s) of immune surveillance, causing cancer cell clearance at the pre-metastatic niche.

## Abstract (web summary)

Exosomes are extracellular vesicles that can favor tumor development and metastasis. Here, the authors show that cancer exosomes may also exert a suppressive function; in fact, exosomes from non-metastatic melanoma cells can lead to the recruitment of patrolling monocytes, which clear cancer cells at the pre-metastatic niche.

## Introduction

Exosomes are 30–150 nm membranous extracellular vesicles (EVs) released by most cells^1^, which are found in biological fluids and play pivotal roles in long-distance intercellular communications^2,3^. Exosomes are derived from the multi-vesicular endosome pathway, through reverse inward budding; however, the term is generally applied to the small EVs and does not discriminate between endosome and plasma membrane derived EVs^4^. Exosomes contain and transfer multiple bioactive molecules including nucleic acids (DNA, mRNA, non-coding RNAs), proteins, and lipids. Typically exosomal membranes are enriched in tetraspanins, such as CD9, CD63, and CD81^5^, and the proteins involved in endocytosis and cargo sorting, such as flotillin and TSG101^6^. By transferring bioactive molecules exosomes alter the function of recipient cells^7^; in particular, cancer cell-derived exosomes have been shown to transfer oncogenic traits from aggressive to indolent cancer cells and to normal cells through the delivery of oncogenic proteins, mRNAs^8^, and miRNAs^9^ that inhibit tumor-suppressive factors, accelerate tumorigenesis, and enable tumor formation^10^. Cancer-derived exosomes also support tumor progression by facilitating angiogenesis, modulating the immune system, and remodeling tumor parenchyma^11–14^. Clinically, circulating EVs isolated from cancer patients have been associated with metastasis or relapse, and therefore could serve as important diagnostic and prognostic markers as well as therapeutic targets^15,16^. The reverse is also true: exosome-assisted transfer of unshielded non-coding RNA from cancer-associated fibroblasts to the cancer cells stimulates pattern recognition response and subsequently tumor progression and therapy resistance^17^. Among exosome-mediated effects, which contribute to metastatic dissemination is proteolysis-dependent matrix remodeling^4,18^ and epithelial-to-mesenchymal transition.

Intercellular communications via exosomes are particularly important for the formation of the metastatic niche where exosomes alter the behavior of diverse cell types including the cells of immune system^19,20^. Exosomes are found in most bodily fluids including blood, urine, and saliva^21^. Recently, it has been established that exosomes released into circulation from the primary tumor generate suitable microenvironments in secondary organs prior to the dissemination of metastases^22,23^. Despite the clear importance of exosomes to cancer progression, mechanisms by which they promote the metastatic niche are extremely complex and not fully understood, with multiple factors at play. Exosome release from hypoxic tumors results in elevated angiogenesis and vascular leakage^24,25^. Exosome also promote coagulation and thus increase adherence of circulating tumor cells^26^. Cancer-derived exosomes are also thought to be involved in the suppression of innate immune responses through mobilization of the myeloid-derived suppressor cells^27^, activation of the tumor-associated macrophages^28^, and neutrophils^29^. In addition, cancer exosomes can cause NK cell dysfunction by exposing NKGD ligands^30^ and hamper adaptive immune responses by repressing antigen-presenting cells and cytotoxic T cells (blocking T cell activation, proliferation, and enhancement of T cell apoptosis)^31^.

Monocytes and macrophages are essential constituents of the metastatic microenvironments^32,33^, where they play either tumor-promoting or tumor-suppressive roles, depending on their activation state (polarization)^34^. Non-classical or patrolling Ly6C^low^ monocytes (PMo) (CD14^dim^ in humans) were initially identified for their ability to remove damaged cells/tissues and resolve the vascular inflammatory response^35,36^. For their survival, PMo require the orphan nuclear receptor Nr4a1 (Nur77). Recently, Nr4a1-positive PMo have been shown to scavenge tumor cells and thus reduce metastasis in the lungs^37^. However, the events that regulate the number of PMo at the metastatic niche remain unknown. Here, we show that exosomes released from non-metastatic melanoma cells (Exo^NM^) are taken up by CD11b^+^ myeloid cells in the bone marrow (BM) and cause a Nr4a1-driven expansion of Ly6C^low^ monocytes, which display elevated levels of integrin-β2 (ITGB2) and CX3CR1 (fractalkine receptor), and Nr4a1 orphan nuclear receptor, which together define PMo^38,39^.

Pigment epithelium-derived factor (PEDF) is known for its potent anti-angiogenic and anti-cancer effects^40^. In melanoma, the loss of PEDF promotes early invasive melanoma growth, ameboid motility, and metastasis^41,42^. PEDF is also implicated in the control of inflammation and macrophage polarization^43^; however, the underlying molecular mechanisms are unknown. Here, we demonstrate that PEDF is present at high levels on the surface of exosomes from non-metastatic melanoma cells and its presence is critical for the activation of an innate immune response and elimination of melanoma metastasis. The events triggered by exosomes involve Nr4a1 induction in BM monocytes precursors, leading to PMo expansion, recruitment, and differentiation of TRAIL-positive tumor-reactive macrophages, which kill and phagocytize the tumor cells. PMo, together with NK cells, are responsible for the diminished metastasis as is shown by immune cell depletion experiments. Our results suggest that pre-metastatic tumors generate triggers of innate immune response(s) such as PEDF, which are delivered to the cells of the immune system by exosomes; the loss of these triggers enables immunosuppression and abrogates the immune clearance of cancer cells leading to metastasis.

## Results

### Non-metastatic exosomes block experimental lung metastasis

**Fig. 1:**
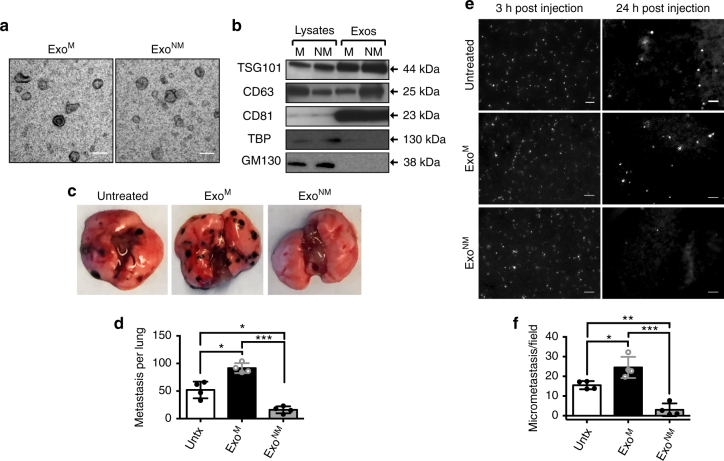
Exo^NM^ inhibit lung colonization by metastatic melanoma. **a** TEM of exosomes isolated by differential ultracentrifugation from metastatic and non-metastatic subcultures of mouse B16F10 melanoma cells. Note similar size and morphology of the Exo^M^ and Exo^NM^ preparations. Scale bar, 50 nm. **b** Western blot of cell lysates and exosomes (Exo) from metastatic (M) and non-metastatic (NM) melanoma cells, probed for ESCRT protein TSG101 and tetraspanins CD63 and CD81, to verify for enrichment in exosome preparation and GM130 and TBP (negative markers). **c** Lung colonization assay: C57BL/6 mice pre-conditioned with Exo^M^ or Exo^NM^ or untreated controls were inoculated intravenously with B16F10 melanoma cells (*n* = 4). Gross images of the tumor burden 2-week post inoculation are shown. **d** Quantification of the lung colonies in **c**. *P < 0.05 and ***P *<* 0.001 by pairwise two-tailed Student’s *t*-test (*n* = 4.). Mean and s.d. values are shown. **e** C57BL/6 mice preconditioned with Exo^M^, Exo^NM^, or untreated controls were inoculated with fluorophore-tagged B16F10 cells. Fluorescence images of the lungs were taken 3 and 24 h after inoculation to assess perfusion and extravasation, respectively. Scale bar, 100 μm. **f** Quantification of extravasated fluorescent B16F10 cells in **e**. *P < 0.05, **P *<* 0.01, and ***P *<* 0.001 calculated by pairwise two-tailed *t*-test; *n* = 4 mice per group, with a minimum of five random images per lung evaluated. Mean and s.d. values are shown

Exosomes produced by metastatic melanoma cells are known to contribute to the formation of the pre-metastatic niche, which leads to colonization by circulating tumor cells^44,45^. We sought to determine whether exosomes from non-aggressive, poorly metastatic melanomas (“non-metastatic” exosomes) could influence the pre-metastatic niche by comparing exosomes from metastatic and non-metastatic variants of the mouse (B16F10) and human (A375) melanoma cell lines (Exo^M^ and Exo^NM^, respectively). Metastatic and non-metastatic variants of mouse (B16) and human (A375) melanoma cell lines were generated by expression of a type 2 tumor suppressor PEDF^46,47^ (see Methods). Exo^M^ and Exo^NM^ display similar size distribution, morphology, and molecular composition, as is shown by transmission electron microscopy (TEM, Fig. 1a), dynamic light scattering, nanotracking analysis (Supplementary Fig. 1a–d), and by Western blotting (Fig. 1b and Supplementary Fig. 2). Exo^M^ and Exo^NM^ contain similar amounts of exosomal markers CD63, CD81, and TSG101, while lacking markers that are typically not found in exosomes (Golgi marker GM130 and the nuclear marker TATA binding protein, TBP), confirming the purity of the isolates. However, these exosomes have dramatically different functional effects on melanoma metastasis. C57BL/6 mice preconditioned with Exo^M^ from B16F10 cells and inoculated with syngeneic B16F10 pigmented melanoma cells (Supplementary Fig. 3a) present with a significant increase in the metastatic burden over the baseline (no pre-treatment). In contrast, pre-conditioning with Exo^NM^ significantly reduces metastasis (Fig. 1c, d). In agreement, athymic nude mice preconditioned with intravenous Exo^M^ injections prior to the tail vein inoculation of fluorophore-tagged A375 melanoma cells display significant metastatic burden after 9 weeks and identical pre-treatment with Exo^NM^ decreases metastasis by ~10-fold (Supplementary Fig. 3b–d). These data demonstrate that “non-metastatic” exosomes have a capacity to inhibit lung metastasis.

Lung colonization by metastasizing cancer cells is influenced by multiple factors, including early events (extravasation, innate immune responses) and late events (survival, proliferation, angiogenesis, and adaptive immune responses)^48^. Seeking the stage at which Exo^NM^ may interfere with metastatic dissemination, we assessed the extravasation of carboxyfluorescein succinimidyl ester (CFSE)-labeled fluorescent B16F10 mouse melanoma cells in immune-competent C57BL/6 mice (Supplementary Fig. 3a). Two hours after inoculation, B16F10 cells are found en masse, presumably in the lung vasculature, regardless of pre-conditioning. After 24 h, extravasation is significantly increased in mice treated with Exo^M^ compared to control. In contrast, there is ~6-fold fewer metastatic colonies in the lungs of mice treated with Exo^NM^ than in Exo^M^ controls (Fig. 1e, f). A similar effect is observed when highly aggressive human melanoma cells (C8161-HA^49^) are injected in athymic nude mice following pre-treatment with exosomes from highly aggressive (C8161-HA) and their natural counterpart, poorly aggressive (C81-61-PA^49^) human melanoma cell lines (Supplementary Fig. 4a, b). A comparable decrease of the metastatic burden in the short-term and long-term models suggests that Exo^NM^ act primarily at an early stage, and analogous responses in immune-competent mice and in athymic nude mice indicate the involvement of innate immune response.

### Exo^NM^ increase the PMo population in the lungs

**Fig. 2:**
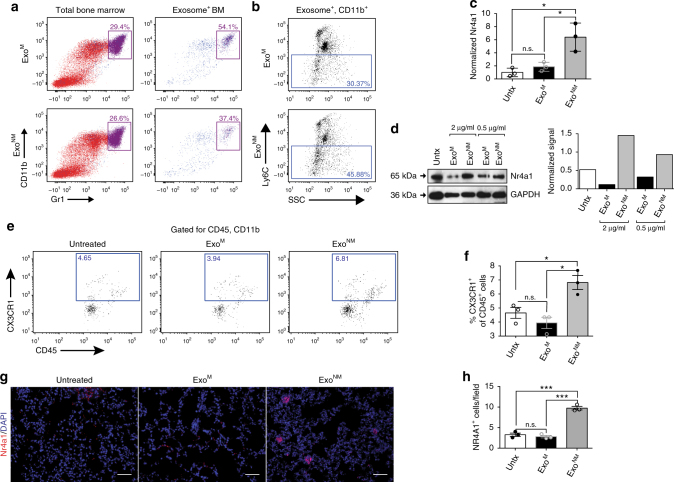
Exo^NM^ increase PMo population. C57BL/6 mice were treated with Exo^M^ or Exo^NM^, prior to BM isolation. **a** Flow cytometry shows the uptake of fluorophore-tagged exosomes by the myeloid cells in the BM. Left: Flow plots of the total CD11b^+^Gr1^+^ BM populations. Right: Plots show a higher percentage of CD11b^+^Gr1^+^ cells among the BM cells positive for exosome uptake. A representative of three independent experiments is shown. **b** Treatment with Exo^NM^ leads to an increase in Ly6C^low^CD11b^+^ cells compared to Exo^M^ in the total myeloid population. **c** qRT-PCR of mRNA encoding Nr4a1. Mouse BM monocytes were treated 24 h with exosomes (10 μg ml^−1^). The experiment was performed in biological triplicates and technical replicates of two. *P < 0.05 by *t*-test in pairwise comparisons. Averages and s.d. values shown. **d** Western blot shows an increase in Nr4a1 in mouse BM monocytes treated with Exo^NM^. Quantification was performed using ImageJ (National Institutes of Health), utilizing plot profile (areas under the peaks) and integrated density measures. A representative of three experiments is shown. **e**–**h** Exosomes increase the presence of PMo in the lung. C57BL/6 mice were treated with 10 μg Exo^NM^ or Exo^M^ 72 and 24 h prior to sacrifice. CX3CR1^+^ cells present in the lung were detected by FACS or in situ IF. **e** Mouse lungs were lavaged, smashed through a strainer and single-cell suspension stained for CD11b, CD45, and CX3CR1. FACS plots were gated for CD11b^+^CD45^+^ cells and CX3CR1^+^ cells quantified. **f** Quantification of the data shown in **a** (percent CX3CR1^+^of total CD45^+^ cells). *P < 0.05 calculated as above. Mean and s.d. values are shown. **g** IF analysis shows the increased abundance of Nr4a1^+^ cells in the lungs of mice treated with Exo^NM^. Scale bar, 100 μm. **h** Quantification of the data in **g**, expressed as number of Nr4a1^+^ cells field^−1^. Scale bar: 50 μm. *n* = 3 mice per group, five images per section. *P < 0.05, **P *<* 0.001, and ***P *<* 0.0005 by pairwise two-tailed *t*-test; *n* = 5 mice per group, >5 random images per lung. Mean and s.d. values are shown

Previous studies show that exosomes home to specific metastatic niches, e.g., the lungs^50,51^. We evaluated the biodistribution of Exo^NM^ and Exo^M^ labeled with fluorescent lipophilic dyes (DiI, DiD). In agreement with previous studies^51^, exosomes home to the lungs and select lymph nodes in tumor-free and in tumor-bearing mice; in mice bearing subcutaneous B16F10 tumors, exosomes are also found in the liver and spleen (Supplementary Fig. 5a, b). Importantly, a large proportion of exosomes homes to the BM (Fig. 2a and Supplementary Fig. 5c). We have noted no significant differences between the distribution patterns of Exo^M^ and Exo^NM^.

To identify target BM cell population(s), C57BL/6 mice were injected intravenously with DiD-labeled exosomes, and BM cells were isolated and stained for the myeloid markers CD11b and Gr1. FACS analysis shows enrichment in DiD-labeled exosomes in the CD11b^+^Gr1^+^ cell population (Fig. 2a). Noteworthy, exosome treatment results in a pronounced shift in the population distribution of the BM: in the total myeloid population, Exo^NM^ cause a significant increase of the Ly6C^low^ subpopulation, and a coordinate decrease in Ly6C^high^ cells (Fig. 2b). Among Ly6C^low^ cells are non-classical (patrolling) monocytes (PMo), which are critical for the resolution of inflammation and tissue damage^52^ and whose anti-metastatic function has been recently discovered^37^. PMo have been shown to rely on the orphan nuclear receptor Nr4a1 for survival^52^. In agreement, Exo^NM^ but not Exo^M^ cause greater than 3-fold increase in Nr4a1 expression in the cultured primary mouse BM monocytes (Fig. 2c, d and Supplementary Fig. 6) compared to the baseline levels.

Furthermore, PMo express high levels of CX3CR1, which mediates their interactions with the vasculature^36,39^ and anti-metastatic activity^37^. This led us to assess the CX3CR1^+^ monocytes in the lungs of mice after pre-treatment with Exo^NM^ or Exo^M^. Flow cytometry analysis reveals low numbers of CD45^+^CX3CR1^+^ cells in the lungs of mice treated with control vehicle or Exo^M^. In contrast, there is a significant increase in CX3CR1^+^CD45^+^ monocytes in the lungs of mice treated with Exo^NM^ (Fig. 2e, f). The results are confirmed by immunofluorescence (IF) staining of the lung sections of mice treated with control vehicle, Ex^M^ or Ex^NM^ for PMo marker Nr4a1 (Fig. 2g, h).

PMo are characterized by their ability to “crawl” along the vasculature in a process which requires the LFA-1 antigen, a complex of CD11a and integrin β2 (ITGB2). Consistent with the shift toward PMo phenotype, Exo^NM^ upregulates ITGB2 in THP-1 monocytes (Supplementary Fig. 7).

### Monocytes are necessary for anti-metastatic action of Exo^NM^

**Fig. 3:**
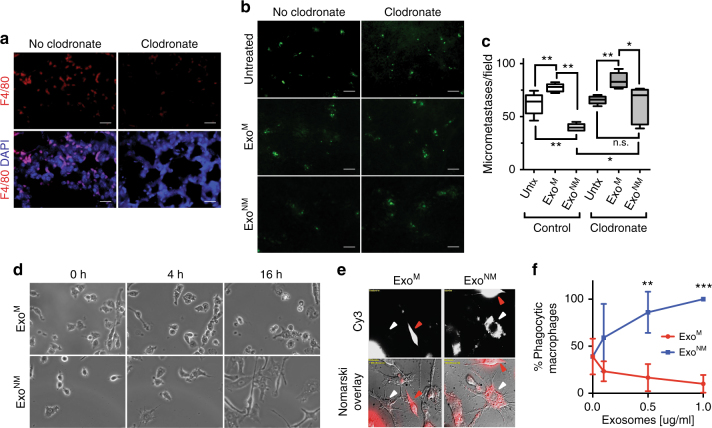
Exo^NM^ act via the cells of monocytic lineage. **a**–**c** Mice were treated with control or clodronate liposomes to eliminate monocytes/macrophages, then pre-conditioned with exosomes (10 μg per mouse) and used in the extravasation assay as described above. **a** Macrophage depletion was assessed by F4/80 IF (*n* = 3, scale bar, 100 μm) or CD11b IF (Supplementary Fig. 5). **b**, **c** The inhibition of B16F10 extravasation by Exo^NM^ was diminished by monocyte depletion. Representative lung images (5× magnification) are shown in **b** (scale bar, 100 μm), and quantitative analysis is shown in **c** (*n* = 5 untreated, *n* = 6 Exo^M^, Exo^NM^, five random images analyzed per animal). *P < 0.05 and **P < 0.01 calculated by two-tailed *t*-test in pairwise comparison. Median values and range are shown. **d** RAW 264.7 macrophages were treated with 10 μg ml^−1^ Exo^M^ or Exo^NM^ and their morphology was assessed by time-lapse microscopy. Representative frames are shown. **e** Engulfment of fluorophore-tagged melanoma cells (red) by RAW264.7 macrophages after treatment with 1 μg ml^−1^ of Exo^M^ or Exo^NM^ (Fluorescence and Nomarski image overlays). **f** Quantification of phagocytosis of the FCSE-tagged melanoma cells by RAW 264.7 macrophages after treatment with Exo^M^ or Exo^NM^ at indicated concentrations. A minimum of three random images were analyzed per condition. **P < 0.01 and ***P < 0.001, respectively, as was calculated by Tukey’s multiple comparison test with Bonferroni post-test. Mean and s.e.m. values are shown

Because melanoma exosomes target cells of the monocytic lineage in the BM (Fig. 2a, Supplementary Fig. 5c), we assessed the contribution of monocytes and/or macrophages to the anti-metastatic effect of Exo^NM^ by measuring tumor cell extravasation in the lungs of mice subjected to monocyte depletion using liposome-encapsulated clodronate^53, 54^. The depletion is confirmed by staining of the lung tissue for the pan-macrophage (F4/80) and myeloid (CD11b) markers (Fig. 3a and Supplementary Fig. 8a, b, respectively). After monocyte depletion, animals were pre-conditioned with exosomes (three injections, 10 μg per animal) and inoculated, via the tail vein, with B16F10 melanoma cells. Consistent with previous observations^44^, lung imaging 24 h post-inoculation shows increased lung colonization in mice pre-conditioned with Exo^M^ compared to the baseline. In contrast, pre-conditioning with Exo^NM^ causes a significant and reproducible decrease in lung colonization. Of note, the decrease in microscopic colonies due to Exo^NM^ is relieved by the clodronate but not by control PBS liposomes (Fig. 3b, c). In contrast, the increased colonization in response to Exo^M^ remains unaffected (Fig. 3b, c). These results suggest a critical role for the cells of monocytic lineage specifically in the anti-metastatic function of Exo^NM^.

### Exo^NM^ induce macrophage differentiation and phagocytosis

Since PMo in some instances differentiate into phagocytic macrophages^55^, we tested whether Exo^NM^ are also capable of inducing macrophage differentiation and tumor cell engulfment. Mouse macrophages (RAW 264.7) were treated with exosomes and their differentiation was assessed as the cumulative macrophage area and length of the processes. Indeed, Exo^NM^ but not Exo^M^ elicits pronounced macrophage differentiation manifested by cell spreading and formation of multiple dendrite-like processes (Fig. 3d; Supplementary Movies 1, 2; and Supplementary Fig. 9a–c). A recent study showed that PMo can control metastasis in part by engulfing cancer cells^37^. In agreement, the exposure of RAW 264.7 macrophages to Exo^NM^ but not Exo^M^ markedly increases their ability to engulf fluorescence-tagged melanoma cells (Fig. 3e, f).

### Exo^NM^ elicit immune response in part via the NK cells

**Fig. 4:**
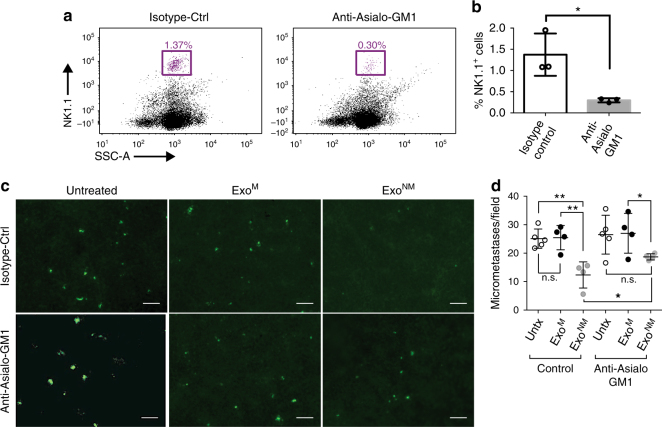
NK cells contribute to exosome-driven tumor cell clearance. **a**, **b** C57BL/6 mice were subjected to NK cell depletion with an anti-asialo GM1 antibody and NK cell depletion assessed by flow cytometry with NK1.1 antibody. **a** Flow plots showing percent NK cells in the spleens of mice 96 h after treatment with isotype control or anti-asialo GM1 antibody. **b** Quantification of the analysis shown in **a**. *P < 0.05 by two-sided *t*-test (*n* = 3). Mean and s.d. values are shown. **c**, **d** Following NK cell depletion, mice were treated with the indicated exosomes and used in an extravasation assay with CFSE-tagged B16F10 cells as described above. **c** Extravasation images taken 24 h after melanoma cell inoculation. Scale bar: 100 μm. **d** Quantification of experiment in **c**. Data are expressed as numbers of extravasated cells per high-powered field (*n* = 4, five random images per animal analyzed for each condition). *P < 0.05 and **P < 0.01 calculated by Tukey’s multiple comparison with (Bonferroni post-test); n.s., non-significant. Median and s.d. values are shown

NK cells play a critical role in preventing metastasis by recognizing tumor cell ligands resulting in the clearance of circulating tumor cells^56^ and PMo have been previously shown to recruit NK cells (by producing CCL3/4/5) to the sites of metastasis where they eliminate cancer cells^37^. To test whether Exo^NM^ induce similar events, we depleted NK cells using pre-treatment with an anti-Asialo-GM1 antibody prior to exosome treatment and tumor cell inoculation (Fig. 4a, b). NK cell depletion significantly reduces the propensity of Exo^NM^ to inhibit lung colonization in the extravasation assay and similar to monocyte depletion experiments, the reversal of Exo^NM^ effects by NK cell depletion is not absolute, suggesting other mechanisms in addition to NK recruitment causing metastasis prevention by Exo^NM^ (Fig. 4c, d).

### Exo^NM^ contain surface PEDF

**Fig. 5:**
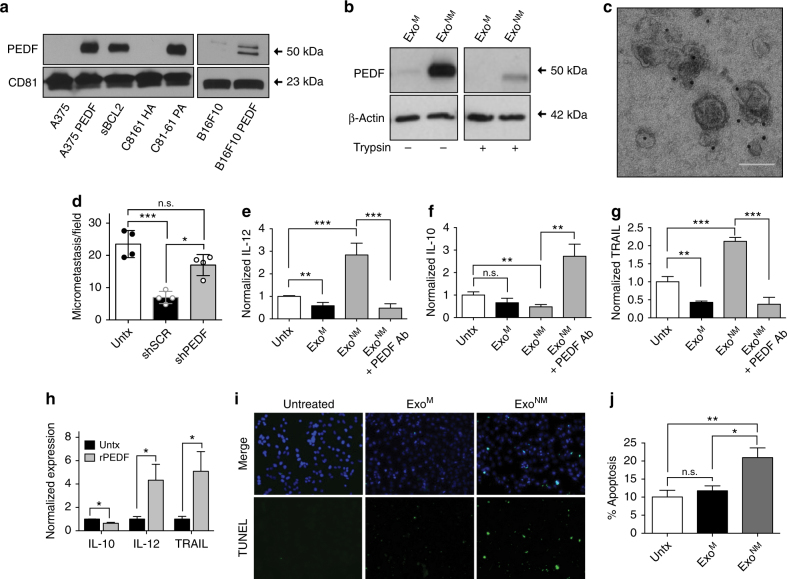
The involvement of PEDF in exosome-driven melanoma cell clearance. **a** Western blot shows PEDF enrichment in exosomes from non-metastatic melanoma cell lines (A375 PEDF, SBcl2, C81-61PA, B16F10 PEDF) and the lack of PEDF in the metastatic exosomes (A375GFP, C8161 HA, B16F10 GFP, and C81-61 PA shPEDF). **b** Limited trypsin digestion indicates that PEDF is tethered to the outer leaflet of exosomal membranes. Exosomal protein is loaded at 10 μg per well. **c** Immunogold staining—TEM of Exo^NM^ confirms PEDF localization to exosome surface. Scale bar, 100 nm. **d** Mice were pretreated with exosomes from non-metastatic C81-61 PA melanoma cells transfected with shPEDF lentiviral vector (shPEDF) or scrambled control shRNA miR (ShScr) (*n* = 4, five random images analyzed per animal for each condition). *P *<* 0.05 and ***P < 0.001 calculated by two-sided Student’s *t*-test in pairwise comparisons. Means, s.d. values are shown. **e**–**g** RAW 264.7 macrophages were treated overnight with vehicle, Exo^M^ or Exo^NM^. PEDF neutralizing antibodies were added to Exo^NM^ where indicated. IL-12, IL-10, and TRAIL mRNA were measured by qRT-PCR (*n* = 6). Three biological and two technical replicates were assessed. **P < 0.01 and ***P < 0.0005 by two-sided *t*-test, n.s., non-significant. Averages and s.d. values are shown. **h** RAW 264.7 macrophages were treated overnight with vehicle PBS or 20 nM recombinant PEDF (rPEDF). IL-12, IL-10, and TRAIL mRNA were measured by qRT-PCR as in **e**–**g**. *P *<* 0.05 calculated by two-tailed *t*-test in pairwise comparison. Averages and s.d. values are shown. **i**, **j** Co-cultures of A375 cells and RAW 264.7 macrophages were treated with exosomes (3 μg ml^−1^) for 24 h and apoptosis measured by TUNEL. Representative images and quantitative analysis are shown. A minimum of 10 fields was analyzed per condition. *P < 0.05 and **P < 0.001 by two-sided *t*-test. Mean and s.d. values are shown

In previous studies by our laboratory as well as other groups, it was established that PEDF renders melanoma cells non-metastatic^42,47^. Importantly, PEDF is also expressed by most non-metastatic cell lines and its expression in patient samples correlates with metastatic dissemination^41,47,57^. To determine if PEDF also plays a role in the exosome-mediated inhibition of metastasis, we assessed the PEDF levels in exosomes harvested from melanoma lines with forced (A375-PEDF, B16F10-PEDF) or endogenous (sBCL2, C81-61-PA)^47^ PEDF expression and from their metastatic counterparts (A375, B16F10, C8161-HA). Of note, all Exo^NM^ in our study display high PEDF content, while Exo^M^ contain little or no PEDF (Fig. 5a and Supplementary Fig. 10a–c) and PEDF enrichment in exosomes correlates with cytoplasmic levels in the source cells (Supplementary Fig. 11a, c). To further confirm that PEDF localizes specifically to exosomes, exosomes from PEDF-expressing A375 melanoma cells were isolated using sucrose gradient ultracentrifugation. PEDF is detected at densities ranging from 1.08 to 1.17 g ml^−1^, where exosomes are concentrated as is confirmed by the presence of exosome marker CD81 (Supplementary Fig. 11b, d). Since PEDF acts via cell surface receptors, its localization within exosomes is critically important. Consistent with surface localization, limited trypsin digestion eliminates most of the exosomal PEDF but not β-actin, which is localized in the exosome lumen (Fig. 5b and Supplementary Fig. 12), suggesting PEDF to be tethered to the outer leaflet of exosomal membrane. The molecular weight of residual PEDF-reactive band on the western blot is reduced (Supplementary Fig. 12) consistent with the cleavage of membrane-tethered protein. To corroborate this finding, immunogold labeling/electron microscopy (EM) was used to directly visualize PEDF localization (Fig. 5c).

In poorly aggressive melanoma cell line C81-61, PEDF is endogenously expressed at high levels in the cytoplasm^46,47^ and in exosomes (Fig. 5a and Supplementary Fig. 13). PEDF knockdown using shRNA significantly reduces exosomal PEDF content (Supplementary Fig. 13) and abolishes their anti-metastatic effect (Fig. 5d). In agreement, treatment with PEDF peptide mimetic attenuates metastatic ability of B16F10 cells (Supplementary Fig. 14).

### Exosomal PEDF causes macrophage polarization/cell killing

Tumor-associated macrophages receive cues from the microenvironment, which determine their tumor-promoting or tumor-suppressive state (polarization)^32^. Tumor-promoting macrophages express higher IL-10 levels, while tumor-reactive macrophages produce predominantly IL-12. We have discovered that Exo^NM^ dramatically increases IL-12 mRNA in cultured macrophages; in contrast IL-12 mRNA is decreased by Exo^M^ treatment (Fig. 5e). In agreement, Exo^NM^ reduces IL-10 mRNA in macrophages (Fig. 5f). Importantly, a PEDF neutralizing antibody reverses the effects of Exo^NM^, while there is no change in Exo^M^ function (Fig. 5e, f). Similar to Exo^NM^, recombinant human PEDF (rPEDF, 1–20 nM) is sufficient to promote macrophage differentiation (Supplementary Fig. 9a–d).

PEDF was also known to upregulate tumor necrosis factor-related apoptosis inducing ligand (TRAIL)^43^. In agreement, macrophage exposure to Exo^NM^ but not to Exo^M^ causes a significant increase of TRAIL mRNA, which is abolished by PEDF neutralizing antibody suggesting PEDF dependence (Fig. 5g). In agreement, rPEDF also alters IL-10, IL-12, and TRAIL expression in RAW 264.7 macrophages in a manner similar to Exo^NM^. Overnight treatment with 20 nM rPEDF led to increases in TRAIL and IL-12 expression and a decrease in IL-10 expression as evidenced by the mRNA levels (Fig. 5h). Moreover, in co-cultures of RAW 264.7 macrophages with A375 melanoma cells, Exo^NM^ potently induce apoptosis of the melanoma cells, but not of macrophages as is ascertained by TUNEL and Annexin V staining (Fig. 5i, j and Supplementary Fig. 15a). In agreement, Exo^NM^ induce apoptosis only in co-cultures of A375 and RAW 264.7 macrophages but not in respective monocultures (Supplementary Fig. 15b, c). Together, our results indicate that exosomal PEDF can promote PMo differentiation to macrophages, as well as macrophage M1 polarization associated with killing and subsequent phagocytosis of melanoma cells.

### Exosomes from patient sera protect against metastasis

**Fig. 6:**
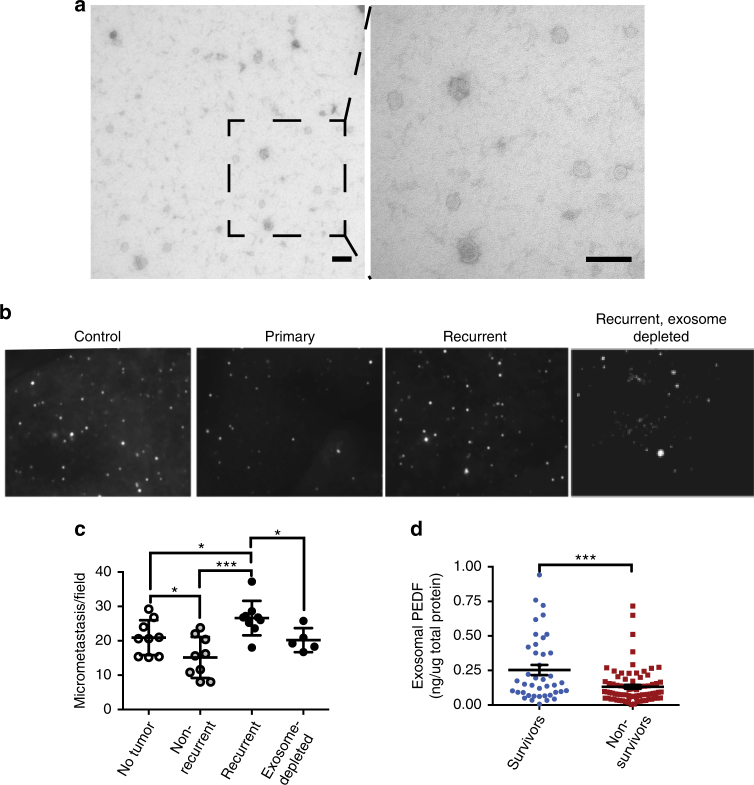
Exosomes from the sera of patients with non-recurrent melanoma suppress lung metastasis. **a** Representative TEM of exosomes isolated from the sera of melanoma patients. Scale bar: 100 nm. **b** Exosomes from the sera of healthy control donors and of patients with primary and recurrent melanoma were injected into athymic nude mice. Exosome-depleted serum from recurrent patients was used as control. Nude mice were injected i.v. three time, with 10 μg exosomes, 48 h apart followed by injection of CFSE-labeled A375 melanoma cells. The extravasated melanoma cells were counted in the lungs 24 h later. **c** Analysis of the data presented in **b**. *P < 0.05, **P < 0.01, and ***P < 0.001 calculated by Tukey’s multiple comparison test with Bonferroni post-test (*n* = 9 for tumor-free, non-recurrent and recurrent groups, *n* = 5 for the group treated with exosome-depleted serum, five random images per animal were analyzed). Mean and s.d. values are shown. **d** Patients were separated into cohorts according to survival (death from melanoma over >5-year follow-up). Exosomal PEDF was measured by ELISA and normalized to the total protein content and correlated to survival. Statistical significance was determined by two-tailed *t*-test. *P* *<* 0.0003. *n* = 41 (survivors) and *n* = 79 (non-survivors). Median and s.e.m. values are shown

The experiments above demonstrate that exosomes from non-metastatic melanoma cell lines can block metastasis by activating PMo. However, it was unclear whether exosomes from patients have similar properties. To determine if exosomes produced by non-metastatic primary melanoma could curtail metastatic spread, we used archival serum samples collected at the time of surgery from patients with primary melanoma. The patients were subsequently stratified on the basis of recurrence after 5-year follow-up (recurrent/metastatic and non-recurrent/non-metastatic). Exosomes were isolated from sera by ExoQuick precipitation followed by affinity spin columns and the preparation quality was assessed by TEM (Fig. 6a) and by Western blot for exosomal markers (Supplementary Fig. 16a–c).

To test the effect of the patients’ exosomes on lung metastasis, nude mice were pre-treated with human exosomes as described above and subsequently received tail vein injections of CFSE-labeled A375 melanoma cells. Of note, serum exosomes from recurrent patients cause a significant increase in the number of lung colonies compared to the “neutral” exosomes from healthy volunteers or exosome-depleted patient serum (Fig. 6b, c and Supplementary Table 1). More importantly, exosomes from the patients with non-recurring tumors cause a statistically significant decrease in lung colonization compared to control exosomes (Fig. 6b, c and Supplementary Table 1), suggesting that our findings using Exo^NM^ from cultured melanoma cell lines reflect the properties of exosomes generated by human melanoma. Of note, we have assessed the correlation between exosomal PEDF levels and survival. Patients were separated into survivors and non-survivors cohorts (*n* = 41 and 79, respectively) with recurrence established in a more than 5-year follow-up. Although there is significant variability in PEDF contents, exosomes from non-survivors present with significantly lower PEDF contents (Fig. 6d). Together, our results indicate that exosomes from the sera of non-metastatic melanoma patients suppress metastatic colonization and this effect is associated with higher PEDF content in serum exosomes.

## Discussion

Over the past few years, there has been an explosion of research focused on exosomes, which led to the discovery that exosomes released from tumor cells interact with a broad range of cell types in distant organ environments allowing for the formation of tumor-promoting pre-metastatic niches^22,23^. Given the importance of exosomes to the interaction between cancer cells and the cells of the microenvironment, we reasoned that exosomes from melanoma cells with varying propensities to metastasize would have contrasting effects on pre-metastatic niche formation. Indeed, while previous data established that exosomes from highly aggressive melanoma cells promote metastasis, in this study, we provide data that demonstrates the anti-metastatic functions of exosomes released from non-metastatic melanoma cells. Moreover, these findings likely reproduce the processes that take place in patients with non-recurrent melanoma and possibly other cancers, because exosomes from archival serum samples taken at the time of surgery from melanoma patients whose tumor did not recur, retained the ability to suppress lung colonization in mouse model.

In a recent study, Hanna et al. discovered the key role of non-classical PMo in the clearance of cancer cells at the site of metastasis, whereby they recruit cytotoxic NK cells leading to eradication of metastasizing cancer cells^37^. We have independently discovered that the clearance of metastasizing cancer cells driven by the “non-metastatic” exosomes stemmed from the expansion of the Ly6C^low^ PMo population. We have traced these exosomes to the CD11b^+^Gr-1^+^ cells in the BM of recipient animals and demonstrated that they induced the Nr4a1 nuclear receptor in BM monocytes causing PMo population expansion and increased presence at the pre-metastatic niche. In agreement with findings by Hanna et al., our in vivo data suggest significant contribution of the NK cells in the anti-metastatic effect of Exo^NM^; however, the in vitro data indicate NK-autonomous effects, which include tumor cell killing by fratricide and engulfment by macrophages. In addition, we observed that non-metastatic exosomes also promote differentiation and polarization of macrophages, which then engage in tumor cell killing and phagocytosis. This sequence of events is summarized in Supplementary Figure 17.

In earlier studies, others and we have demonstrated that in melanoma, the switch to a highly aggressive and invasive metastatic state is associated with the loss of PEDF, a type 2 tumor suppressor^41,42,47,58,59^. Importantly, we were able to link the anti-metastatic, pro-immune properties of melanoma exosomes with PEDF, whereby tumor cells that lost PEDF expression no longer produce exosomes that maintain cancer surveillance by the innate immune system. This is the first study, which implicates cancer-derived exosomes in the induction of cancer immune surveillance and clearance.

Importantly, our results support the opposing roles of classical and non-classical (patrolling) monocytes in cancer. On the one hand, classical (inflammatory) CCR2^+^CX3CR^−^Ly6C^high^ monocytes (IMo) play a key role in the development of metastasis whereby they differentiate into inflammatory macrophages, which populate the pre-metastatic niche and support extravasation, survival, and proliferation of metastatic cancer cells^60^. Conversely, CCR2^−^CX3CR^+^Ly6C^low^ PMos are enriched in the microvasculature of tumor-challenged lung and reduce tumor metastasis^37^. In contrast to classical monocytes (Imo), PMo rarely extravasate from the vasculature under normal circumstances; instead, they scour microvessels for the cell debris and particles^36^. In the presence of tumor cells, their patrolling behavior is disrupted and replaced by accumulation at the tumor site. Hanna et al. show that PMo recruitment to the tumor site is mediated by CX3CR1, which interacts with endothelial-derived CX3Cl1. However, the same study underscores the low abundance of PMo in the lung. Our study provides a new mechanism underlying the increased PMo presence at the sites of metastasis, which is particular for pre-metastatic tumors. We discovered that early-stage, pre-metastatic melanoma express trigger(s) of immune clearance which are loaded onto exosome surface and delivered, to the monocyte progenitors in the BM of the host causing the expansion of CX3CR^+^Ly6C^low^ monocyte population.

In conclusion, our study provides a completely new mechanism responsible for the increased presence of PMo at the pre-metastatic niche and continuous elimination of circulating tumor cells in the tumor-bearing host. On the other hand, we for the first time demonstrate that prior to the acquisition of the metastatic capacity, tumors continuously alert host immune system by producing exosomes, which carry trigger(s) of innate immune responses and we identify one such trigger, PEDF. Furthermore, analysis of exosomes isolated from archival serum samples corroborates these findings. Taken together, our result point toward a potential new type of cancer immunotherapy based on the use of vesicular structures for delivery of such immune trigger. Interestingly, both previous studies and our current results demonstrate the anti-metastatic role and therapeutic potential of PEDF in melanoma^47,61–64^ and other cancers^65–68^. Although PEDF-derived peptide shows only mild anti-metastatic activity in our melanoma model, this could potentially be improved by its loading onto exosomes to increase targeting to the immune system and thus facilitate the development of PEDF-derived peptides into effective immunomodulatory, anti-metastatic therapy.

## Methods

### Cell culture

B16F10 melanoma cells (ATCC, Manassis, VA) were cultured in Dulbecco’s Modified Eagle Medium (DMEM) containing 5% fetal bovine serum (FBS) and 1% penicillin/streptomycin. A375 melanoma cells (ATCC) were cultured in DMEM containing 10% FBS and 1% penicillin/streptomycin. sBCl2 cells were a gift from Dr. Meynard Herlyn (Wistar Institute) and C8161/C81-61 cells were a gift from Dr. Mary J.C. Hendrix (Stanley Manne Children’s Research Institute) and cultured in 50/50 DMEM/F12 containing 5% FBS, 1% penicillin/streptomycin, and RPMI in 10% FBS, 15 penicillin/streptomycin, respectively. PEDF was overexpressed in A375 and B16F10 melanoma cells using lentivirus encoding full-length human PEDF cDNA inserted between XbaI and BamHI sites within the prrl.CMV.EGFP.wpre.SIN lentiviral vector^41^. PEDF knockdown in C81-61 melanoma cells was performed using shRNA-mir in pGIPz lentiviral vector VL2HS_221662 (Open Biosystems, Huntsville, AL). In brief, lentiviruses were generated by co-transfection into HEK 293T cells using the Lenti-X HT packaging system. Culture supernatants were collected, concentrated, titrated, and used for cellular transduction. Cells stably expressing the construct were selected for using puromycin, then identified and sorted for GFP expression.

RAW macrophages (ATCC, Manassis, VA) were cultured in DMEM containing 10% FBS and 1% penicillin/streptomycin. Cells were passaged approximately every 3 days. Primary mouse macrophages were isolated from the BM as described below and cultured in 1/1 DMEM/F12 media supplemented with 10% FBS, 1% penicillin/streptomycin, and 20 ng ml^−1^ of M-CSF (Biolegend, San Diego, CA).

All cell cultures were maintained in 5% CO_2_ at 37 °C. All cell lines were authenticated using MDACC Cell Bank Services and tested for mycoplasma contamination using MycoAlert™ Mycoplasma Detection Kit (Lonza, Walkersville, MD) on a monthly basis. For all cell lines, frozen aliquots of 10^6^ cells were stored at a passage below 5 and a fresh aliquot used after 10 consecutive passages.

### Isolation of BM macrophages

Mouse BM-derived macrophages were isolated and matured as described below^69^. Briefly, C57BL/6 mice were sacrificed and the hind limbs were removed using aseptic technique, leaving the femur and tibia intact. The muscle was removed from the bones with a razor blade. The bones were then cut at the joints and the BM flushed with sterile DPBS (calcium and magnesium free) using 1 ml syringe with a 27-gauge needle. BM cells were centrifuged at 500 × *g*, re-suspended in 50/50 DMEM/F12 media supplemented with 10% FBS, 1% penicillin streptomycin and 20 ng ml^−1^ of M-CSF (Biolegend) and plated at 4 × 10^5^ cells per 10 cm tissue culture dish. BM cells were incubated at 37 °C, 5% CO_2_ for 3 days at which point they were supplemented with additional 5 ml of cell culture medium containing 20 ng ml^−1^ of M-CSF. Cells were allowed to grow for an additional 4 days, harvested and used for further studies.

### Experimental animals

Mice were housed and maintained at the Northwestern University Center for Comparative Medicine, according to the NIH guidelines and following protocols approved by the Northwestern University Animal Care and Use Committee. Female athymic *nu/nu* mice (4–6 weeks of age) were from Harlan Laboratories and female C57BL/6 mice (4–6 weeks of age) were from Jackson Laboratories. Age-matched female mice were used for all the studies and the group sizes calculated to achieve 80% power (see statistical methods below).

### Exosome collection and characterization

Melanoma exosomes were isolated from conditioned media (CM) by differential ultracentrifugation^70^. In brief, cells were cultured in DMEM containing 10% exosome-depleted FBS (Life Technologies) and 1% penicillin streptomycin for 72 h. The CM was collected and centrifuged at 2000 × *g* to remove dead cells and debris. Next, larger vesicles and finer debris were removed by centrifugation at 10,000 × *g* for 30 min. Exosomes were then centrifuged at 100,000 × *g* for 70 min on top of a 30% sucrose cushion, and washed in PBS by another centrifugation step (100,000 × *g*, 70 min). Exosomes were re-suspended and stored in PBS. Protein content of exosomes was measured by BCA Protein assay (Thermo Scientific) and treatment doses were based on exosome protein concentration. Exosome size, morphology, and number were characterized using dynamic light scattering (Malvern), NanoSight imaging, and TEM (FEI Spirit G2 TEM). Exosome counts were determined in each nanotracking experiment and exosome number per μg protein calculated. For exosome tracing experiments, the lipophilic di-alkyl indocarbocyanine dyes, DiI or DiD (Life Technologies), were added to exosome preparations at a concentration of 2.5 μM after the first 100,000 × *g* ultracentrifugation step. The labeled exosomes were then washed twice in PBS by pelleting the exosomes and discarding the supernatant.

### Exosome isolation by sucrose gradient ultracentrifugation

The exosome pellet isolated after the final 100,000 × *g* spin as outlined above was resuspended in 1 ml of 2.5 M sucrose, 20 mM HEPES (pH 7.4), and loaded on the bottom of a sucrose gradient over the concentrations of 2.5 to 0.25 M sucrose. Exosomes were spun for 24 h at 100,000 × *g* after which 10 fractions were collected from the top, diluted in 16 ml PBS, and spun for 1 h at 100,000 × *g* for 1 h. The pellets were collected in 50 μl PBS and analyzed by Western blotting.

### Exosome isolation form human serum

Due to the low volumes of sera available from patients, we isolated exosomes using combinations of commercial reagents. Serum was first filtered via 0.45 μm syringe filters and exosomes were precipitated using ExoQuick reagent (System Biosciences), according to the manufacturer’s protocol. Membrane affinity spin columns (exoEasy Maxi, Qiagen) were then used to eliminate non-exosomal serum contaminants.

For PEDF analysis, ExoQuick-precipitated exosomes were purified using CD63/CD81 conjugated Dynabeads (Invitrogen). The protein contents were measured using BCA assay and normalized to 100 μg ml^−1^. Exosomes were then characterized using TEM and western blotting as outlined above.

### SDS-PAGE and western blotting

For western blots, 20 μg of total protein extract for cell lysates or 10 μg exosomal protein were resuspended in 4× Laemmli sample buffer (Biorad) and incubated at 95 °C for 5 min. The protein samples were then resolved on Tris/Glycine/SDS pre-cast polyacrylamide gels (PAGE, a 4–20% gradient, Bio-Rad, 30 min at 200 V). Proteins were transferred onto polyvinylidene fluoride membranes (wet transfer, 2 h, 40 V). The membranes were blocked in 5% milk in Tris-buffered saline containing 0.1% Tween 20 (TBST) for 1 h. The membranes were incubated with the primary antibodies (Supplementary Table 2) in blocking buffer overnight at 4 °C, washed 3 × 10 min in 0.1% TBST and incubated with the appropriate HRP-conjugated secondary antibody in blocking buffer for 1 h at room temperature. The membranes were then washed in 0.1% TBST (3 × 10 min) and developed with Amersham ECL Western Blotting Detection Reagents (GE Healthcare). For each cropped blot image, a supplementary figure with uncropped blot with molecular weight marker is provided.

### RT-PCR

RAW 264.7, THP-1, or BM-derived macrophages were treated with Exo^M^ or Exo^NM^ for 48 h. mRNA was isolated using an RNeasy kit (Qiagen), and 1 μg of RNA was converted to cDNA using iScript cDNA synthesis kit (Bio-Rad). Quantitative RT-PCR was then used to amplify and measure the cDNA content using PerfeCTa SYBR Green mastermix (Quanta) on a CFX Connect RT-PCR Detection System (Bio-Rad) (for primers, see Supplementary Table 3).

### Flow cytometry

For analysis of the BM cell populations, C57BL/6 mice were treated with Exo^NM^ and Exo^M^, hind limbs collected and BM cells flushed from tibias and fibulae as described above. Lymphocytes were then separated by centrifugation at 500 × *g* after layering on a Histopaque 1.077 g ml^−1^ density gradient. The cells at the interphase were collected and residual red blood cells lysed in ACK lysis buffer for 10 min at 4 °C. Lymphocytes were counted and resuspended in FACS buffer (DPBS containing 1% bovine serum albumin (BSA), 0.1% sodium azide) at 10^7^ cells ml^−1^.

For analysis of lung monocytes/macrophages lungs were lavaged with DPBS, excised, and smashed in DPBS through a 70-μm-cell strainer. Red blood cells were lysed in ACK buffer and the cells washed in FACS buffer, counted and resuspended in FACS buffer (10^7^ cells ml^−1^).

For immunostaining, 100 μl of the cell suspension were blocked in Fc Block (BD Pharmingen) for 20 min at room temperature. The cells were incubated with fluorophore-conjugated antibodies (Supplementary Table 2) for 1 h at 4 °C and washed in FACS buffer. Fluorescence was assessed on a BD LSR Fortessa Analyzer (Robert H. Lurie Comprehensive Cancer Center Flow Cytometry Core) and data were analyzed using FCS Express (De Novo Software).

### Immunohistochemistry

C57BL/6 mice were sacrificed and the lungs perfused with 10% formalin, excised and allowed to fix in formalin for 24 h. The lungs were paraffin embedded and sectioned at the Robert H. Lurie Comprehensive Cancer Center Pathology Core. Five-μm sections were deparaffinized in two changes of xylene, 10 min each. Sections were hydrated by sequential incubations in ethanol, 100% (2 times), 95, 70, and 50%, 5 min each. Samples were then washed in deionized water and incubated with 200 μg ml^−1^ proteinase K (Abcam). For staining, sections were washed in PBS containing 0.1% tween 20 and blocked in PBS with 1% BSA, 1% donkey serum, and 0.3% triton-X100 and 0.01% sodium azide for 1 h at room temperature. Primary antibodies (Supplementary Table 2) were diluted in blocking buffer and incubated overnight at 4 °C. Samples were washed 3 × 10 min in 0.1% tween 20 in PBS and incubated for 1 h with fluorophore-conjugated secondary antibodies in blocking buffer. Slides were then washed three times in PBS with 0.1% tween 20, counterstained with DAPI and mounted in Fluoromount-G (Southern Biotech). The sections were analyzed in a blinded manner, prior to data analysis.

### TEM and immunogold labeling

Exosomes isolated as described above were stained with uranyl acetate^70^. Briefly, exosomes resuspended in 2% paraformaldehyde were allowed to adsorb onto formvar carbon-coated EM grids (Electron Microscopy Sciences) for 20 min. The grids were then washed in 100 μl PBS, fixed in 1% glutaraldehyde for 5 min and washed with water 7 × 2 min. To contrast the samples, each grid was transferred to a 50-μl drop of uranyl-oxalate (1:1 mix of 4% uranyl acetate and 0.15 M oxalic acid) at pH 7 for 5 min. The grids were then embedded in 2% methylcellulose, 4% uranyl acetate (9:1 v:v ratio), for 10 min on ice. Grids were removed from embedding solution with micro-forceps and excess fluid blotted on Whatman no. 1 filter paper. Grids were allowed to air dry for 10 min and stored in grid boxes. Exosomes were visualized using a FEI Tecnai Spirit G2 120 kV TEM.

For immunogold labeling, after paraformaldehyde fixation and adsorption onto EM grids, exosomes were washed 3 × 3 min in PBS 3 and incubated 3 min in 50 mM glycine in PBS to quench free aldehyde groups. Next, exosomes were blocked 10 min with 5% BSA in PBS. PEDF antibody (BioProducts MD) was diluted in 5% BSA at 10 μg ml^−1^ and layered on the EM grids for 30 min. Grids were washed 6 × 3 min with 0.1% BSA in PBS. Protein-A gold conjugates (Ted Pella, Inc.), diluted 1:200 in blocking buffer and incubated on EM grids for 20 min. The grids were washed with PBS 8 × 2 min, followed by 5 min fixation with 1% glutaraldehyde and 8 × 2 min washes in water. Grids were then contrasted, embedded, and images taken as described above.

### Limited trypsin digestion

Exosomes were incubated with 0.05% trypsin for 5 min at room temperature to digest surface proteins only. After digestion, 4× Laemmli buffer was added to the samples, proteins resolved by gel electrophoresis and western blotting performed with antibodies for PEDF and β-actin (luminal marker).

### Phagocytosis assay

RAW 264.7 macrophages and A375 melanoma cells expressing GFP tag were seeded in 24-well plates at 30:1 ratio. After 24 h, Exo^M^ or Exo^NM^ were added to the cells at 0.01, 0.5, and 2.0 μg ml^−1^. Cells were live imaged at 48 h. Before imaging, cover glass was removed and placed in Attoflour® Cell Chamber (Life Technologies) filled with 1 ml of cell superfusion buffer (0.35 mM Na_2_HPO_4_, 110 mM NaCl, 0.44 mM KH_2_PO_4_, 5.4 mM KCl, 1 mM MgSO_4_, 1.3 mM CaCl_3_, 25 mM HEPES, and the pH was adjusted to 7.4). The cell chamber was placed on the microscope platform for imaging. Nomarski/DIC images and confocal images were obtained using the Zeiss AxioVert 200 inverted fluorescence microscope, AxioCam camera, and AxioVision software. Cells were imaged using a 63× objective (N.A. 1.4; oil). The process length and the macrophage surface area were quantified using ImageJ. Images were taken randomly and analyzed in a blinded manner. In an independent experiment, cells were plated in glass bottom dishes treated with exosomes at indicated concentrations and cells were imaged every 15 min for 16 h.

### Apoptosis assays

Terminal dUTP nick-end Labeling Assay (TUNEL): The cells were grown on glass coverslips coated with 0.1% gelatin (45 min at 37 °C). RAW 264.7 macrophages were grown for 24 h in co-culture with A375 melanoma (5:1 ratio) or as monocultures. Exosomes were then added at a concentration of 3 μg ml^−1^. The cells were incubated for additional 24 h, then fixed in 4% paraformaldehyde and permeabilized with 0.1% TritonX-100 for 2 min at 4 °C. Apoptotic cells were detected and apoptotic cells detected using in situ Cell Death Detection Kit (Roche) and visualized by fluorescence microscopy. Images were taken and evaluated in a blinded manner prior to data analysis using Nikon Elements software (Melville, NY, a minimum of eight images per condition).

Annexin V staining: Cells were grown as above, harvested and stained in suspension using Annexin V/propidium iodide apoptosis detection kit (Biolegend). For cell type-specific detection of cell death, the cells were co-stained with antibody for macrophage marker F4/80 (Brilliant Violet Fluorophore) and analyzed by flow cytometry.

### Macrophage differentiation assay

RAW 264.7 macrophages (3 × 10^5^) were plated in 35-mm glass-bottom dishes, allowed to adhere for 24 h and treated with Exo^M^ or Exo^NM^ at a concentration of 3 μg ml^−1^. Images were taken every 15 min for 16 h in a Nikon Biostation (5% CO_2_ at 37 °C) focusing on the formation of dendrite-like projections. For quantification studies the cells were seeded in 6-well dishes. NIS-Elements AR 4.00.03 (Nikon) was used for analysis following imaging. The z-stacks were merged, ROI’s selected, and the surface area and the multi-point function were used to measure area and length, respectively.

### Lung colonization assay

C57Bl6 mice were randomly assigned into groups and injected intravenously with 10 μg (2.6 × 10^8^ particles) Exo^M^ or Exo^NM^ 3 times 48 h apart. Untreated control (100 μl vehicle PBS) was included in each experiment. Last exosome treatment was followed by intravenous inoculation of 10^5^ B16F10 melanoma cells. The mice were sacrificed after 14 days and lung metastasis counted in a blinded fashion. To assess the direct effect of PEDF peptide mimetic mice were given daily injections of peptide reconstituted in vehicle PBS (Adp-Sar-YNLYRVRS-NH2, synthesized to order at peptide synthesis facility at Northwestern University, Simpson-Querrey Institute for BioNanotechnology, Chicago, IL, and supplied at 95% purity). The peptide was used at a final dose of 100 mg kg^−1^.

Athymic nude mice (Harlan) were injected intravenously with 20 μg (2.6 × 10^8^ particles) of Exo^M^ or Exo^NM^ 3 times 48 h apart. After the exosome treatment, GFP-tagged A375 melanoma cells (3 × 10^5^ cells/mouse) were injected via the tail vein. Tumors in the lungs were allowed to develop for 9 weeks at which point the mice were sacrificed, lungs were harvested, and images taken using a Nikon OV-100 imaging station (Northwestern University, Center for Advanced Microscopy). Images were evaluated in a blinded manner prior to data analysis, using Nikon Elements software.

### Lung extravasation assay

Extravasation was assessed in a modified lung colonization assay^41^. Female C57BL/6 mice were randomly assigned into groups and pretreated with exosomes (10 μg/mouse, two times 48 h apart) or vehicle PBS. After the second exosome treatment, the mice were inoculated with 10^6^ B16F10 melanoma cells labeled with CFSE fluorescent dye. Mice were sacrificed at 3 and 24 h after tumor cell inoculation, the lungs excised and fluorescence-labeled cells were visualized with a Nikon AZ-100 fluorescent microscope (Northwestern Center for Advanced Microscopy) or Nikon Diaphot 2000 with 3× objective. Alternatively, the extravasation of human melanoma cells was analyzed following the same protocol. For these experiments, exosomes were isolated from highly aggressive and poorly aggressive C8161 (C8161 HA or C81-61 PA, respectively). Images were taken and evaluated in a blinded manner prior to data analysis using Nikon Elements software (>5 per condition).

### Clodronate depletion of macrophages and monocytes

C57BL/6 mice were depleted of macrophages and monocytes using clodronate containing liposomes^54^. In brief, mice were given intravenous injections of clodronate or control PBS liposomes (200 μl/mouse), 96 and 48 h prior to exosome treatment and inoculation of CFSE-tagged melanoma cells and assessment of extravasation (see above). Six mice in each group were sacrificed and the lungs stained for F4/80 and CD11b, to ascertain the depletion of monocytic cells.

### Depletion of NK cells with anti-asialo GM1 antibody

C57BL/6 mice were pre-treated with anti-asialo GM1 antibodies or non-specific isotype control antibodies. 20 μl anti-asialo GM1 antibodies (Wako Chemicals) were diluted 5× in PBS and were injected intraperitoneal 24 h prior to exosome treatment and extravasation assay. Staining splenocytes with an anti-NK1.1 antibody and analyzing using flow cytometry was used to assess NK cell depletion.

### Functional analysis of patient exosomes

Exosomes from melanoma patients and healthy controls were isolated from serum collected at the New York University School of Medicine following protocol i10362 approved by the Institutional Review Board and carried out in accordance with approved guidelines. All patients provided informed consent prior to serum collection. Melanoma patient serum samples were completely de-identified prior to transfer to Northwestern University. De-identified samples were separated into three groups, healthy control, patients with primary melanoma and no recurrence, and patients with recurrence after resection of the primary melanoma. Melanoma recurrence was determined after a minimum of 5-year follow-up. Patient data are outlined in Supplementary Table 1. For the depletion of exosomes, sera of recurrent melanoma patients were ultra-centrifuged at 100,000 × *g* for 24 h. After ultracentrifugation, the supernatant was removed and used for analysis.

For functional analysis of patient exosomes, to determine the function at promoting or suppressing metastasis 10 μg of exosomes (based on protein content) (isolated using protocol outlined above) was injected i.v. twice, 48 h apart. Following the second exosome dose, 10^6^ CFSE-labeled A375 human melanoma cells were inoculated i.v. Mice were sacrificed, lungs dissected, and the number of melanoma lung colonies was determined by fluorescent microscopy. Exosomes from one patient were tested in three mice.

### Statistical analysis of the quantitative results

Microscopy and flow cytometry results were analyzed in a double-blinded fashion, using ImageJ and FCS Express, respectively. All experiments were repeated at least three times and representative experiments of three are shown. For microscopy data we used at least three biological and five technical replicates. GraphPad Prism version 6 (GraphPad, San Diego, CA) was used for all statistical analysis. Data are presented as means, using standard deviation (s.d.) or standard error meaning (s.e.m.) of at least triplicate measurements, as a measure of sample variance. Mean values ± s.d., *P-*values, and sample sizes are reported in figures and figure legends. Differences between groups/populations were analyzed using Student’s *t*-tests for normal data sets and using non-parametric test (Mann–Whitney) for non-normal data sets. For more than two groups of data we were using Tukey’s multiple comparison test followed by Bonferroni post-test. Normality of the data sets was determined by Kolmogorov–Smirnoff test. The differences were set as significant at *P* ≤ 0.05.

Group sizes were determined using power analysis, based on pilot experiments and data variance (standard deviation). We conservatively assume that exosomes increase or decrease the mean colony number by at least 50% while compared to the untreated control with a common standard deviation of 25% (an effect size of 1.25). To achieve 80% power, a minimum of three mice per group is required. Similar calculations were performed for each experiments based on sample variance and group size adjusted accordingly. We have used no less than four animals per group in all experiments and each experiment repeated at least three times with similar results. Animals were excluded from the study if no tumor take (zero lung colonies) was observed during data collection.

### Data availability

All relevant data are placed within the article and Supplementary Files, or available from the authors upon request.

## Electronic supplementary material

Supplementary Information

Peer Review File

Description of Additional Supplementary Files

Supplementary Movie 1

Supplementary Movie 2
