# Supplementary material for: Pre-metastatic cancer exosomes induce immune surveillance by patrolling monocytes at the metastatic niche
Source: Nat Commun. 2017 Nov 6;8:1319. doi: 10.1038/s41467-017-01433-3 (PMC5673063; doi:10.1038/s41467-017-01433-3)
Supplement: Supplementary file 1 — Supplementary Information [file 41467_2017_1433_MOESM1_ESM.pdf]

Supplementary Material:

Pre-metastatic cancer exosomes induce immune surveillance by patrolling monocytes at the metastatic niche

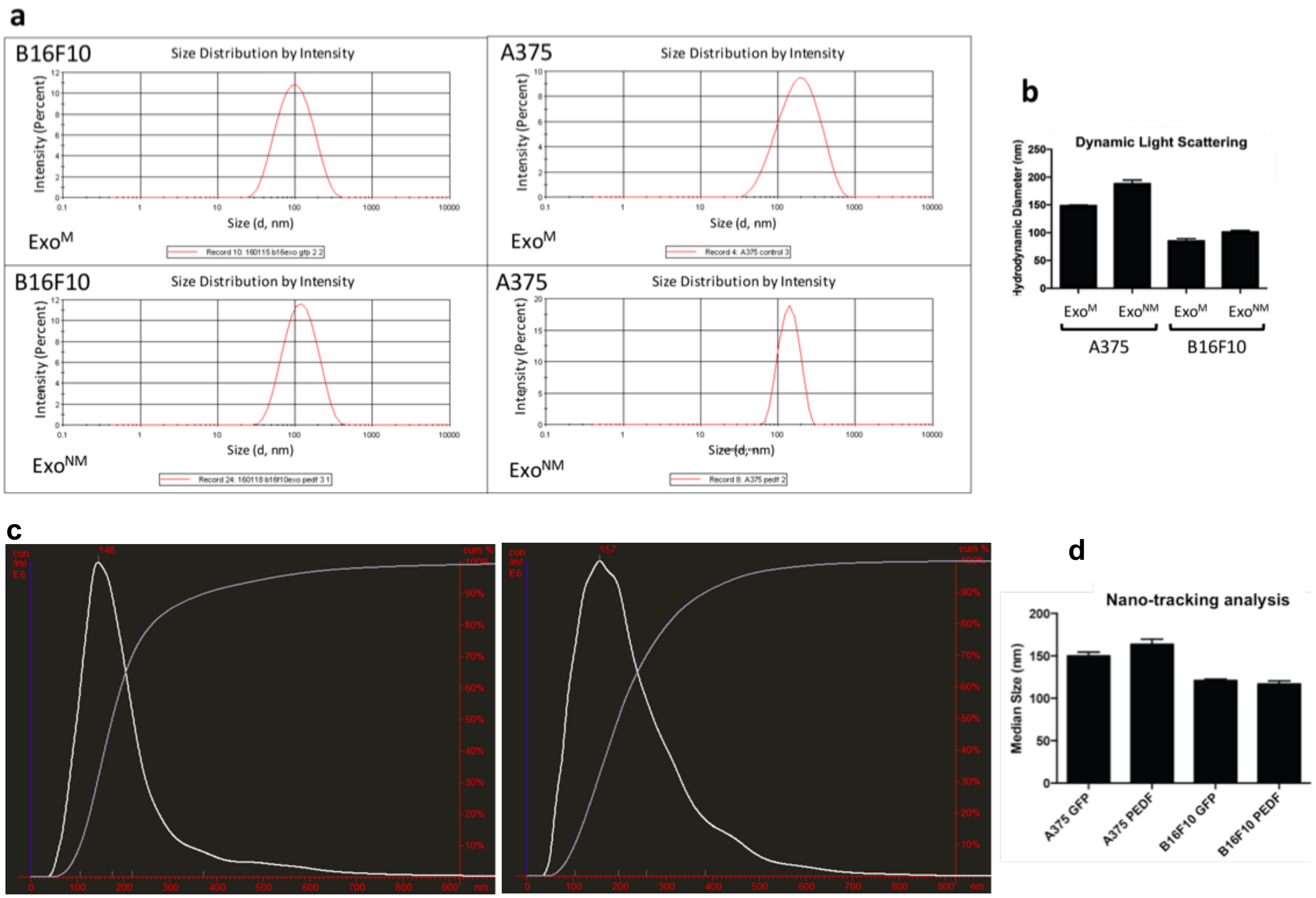

Supplementary Figure 1. Characterization of metastatic and non-metastatic melanoma exosomes. (**a**) Hydrodynamic diameter analysis of exosomes from metasatatic and non-metastatic A375 and B16F10 melanoma cells by dynamic light scattering (DLS). (**b**) Quantification of average hydrodynamic diameter analyzed in ***a***. (**c**) Nano-tracking analysis (Nanosight) showing size of exosomes from metasatatic and non-metastatic A375 and B16F10 (**d**) Quantification of nano-tracking analysis (Nanosight) data acquired in ***c***. Average vaules and s.e.m. are shown.

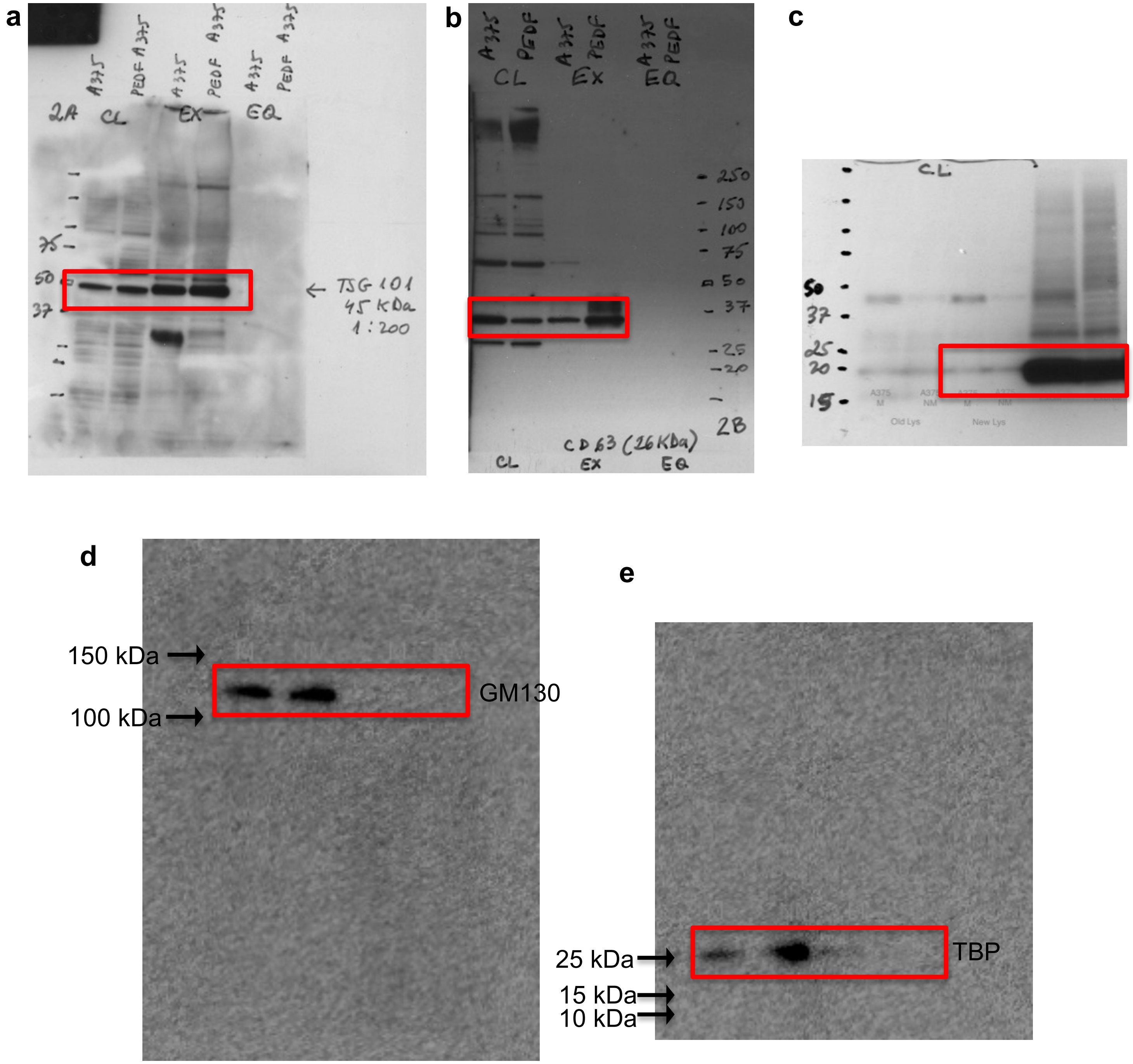

Supplementary Figure 2. Full-size Western blots of melanoma exosomes. **(a)** enrichment for TSG101, late endosome marker (**b, c**) CD63, and CD81, exosome markers (**d, e**) Golgi marker GM130 and nuclear protein TATA binding protein, respectively (used as negative controls). Cell lysates and exosomal protein were loaded at 30 and 10 μg per well, respectively. Images in ***d*** and ***e*** were acquired using Azure C300 chemiluminsecence imaging system.

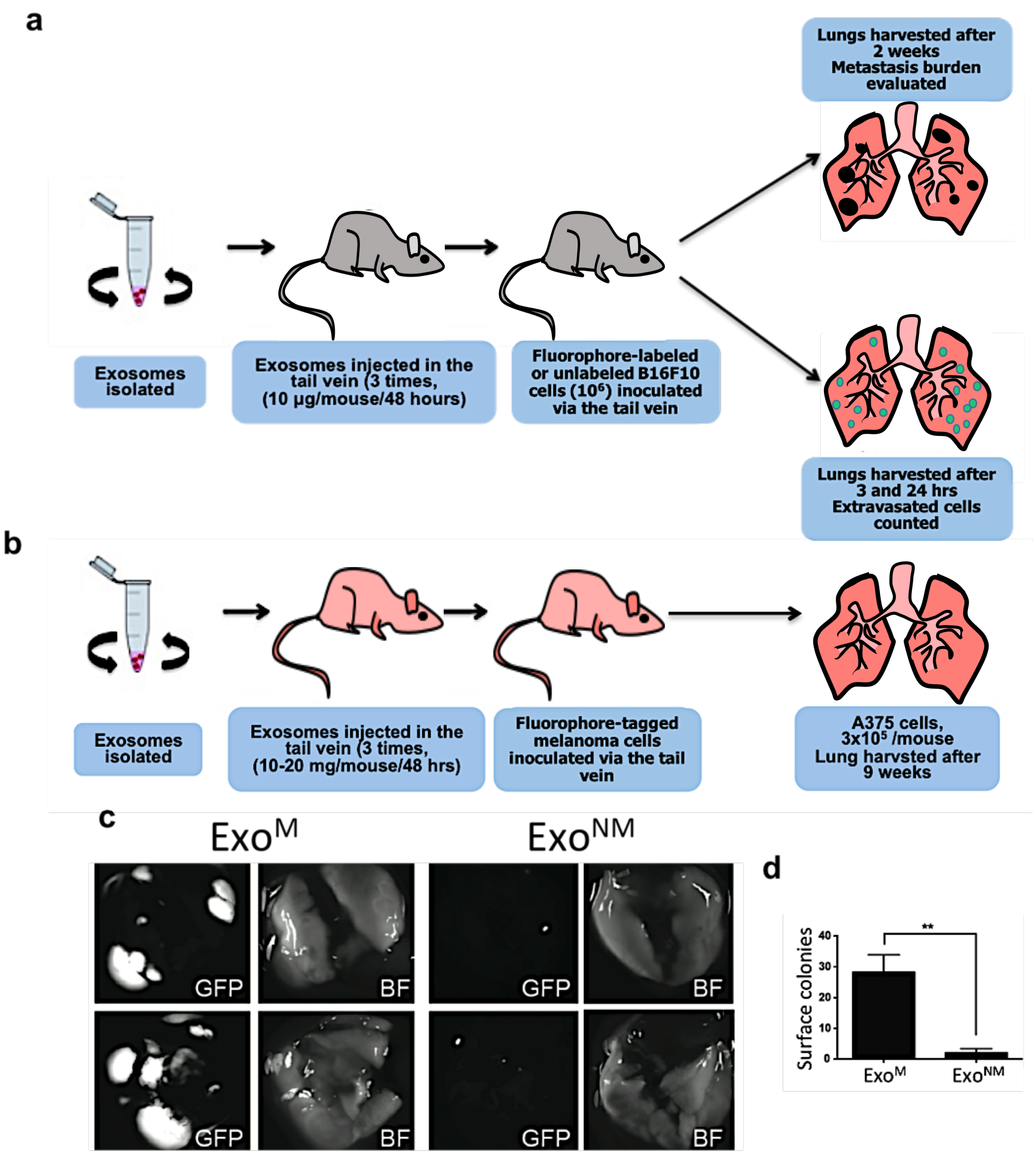

Supplementary Figure 3. The schematics of melanoma *in vivo* experiments. (a) The experiments performed with mouse B16F10 cells in immune competent C57BL/6 mice. **(b)** The diagram of the long-term experiments using human melanoma cells and exosomes (performed in nude mice). **(c)** Representative images of the lungs of mice pre-treated with exosomes and inoculated with fluorophore-tagged A375 cells. **(d)** Quantitative analysis of the experiment in ***c***. n=6, ** indicates *P<0.01* calucated by two-sided Student's *t*-test. Mean abd s.e.m. values are shown.

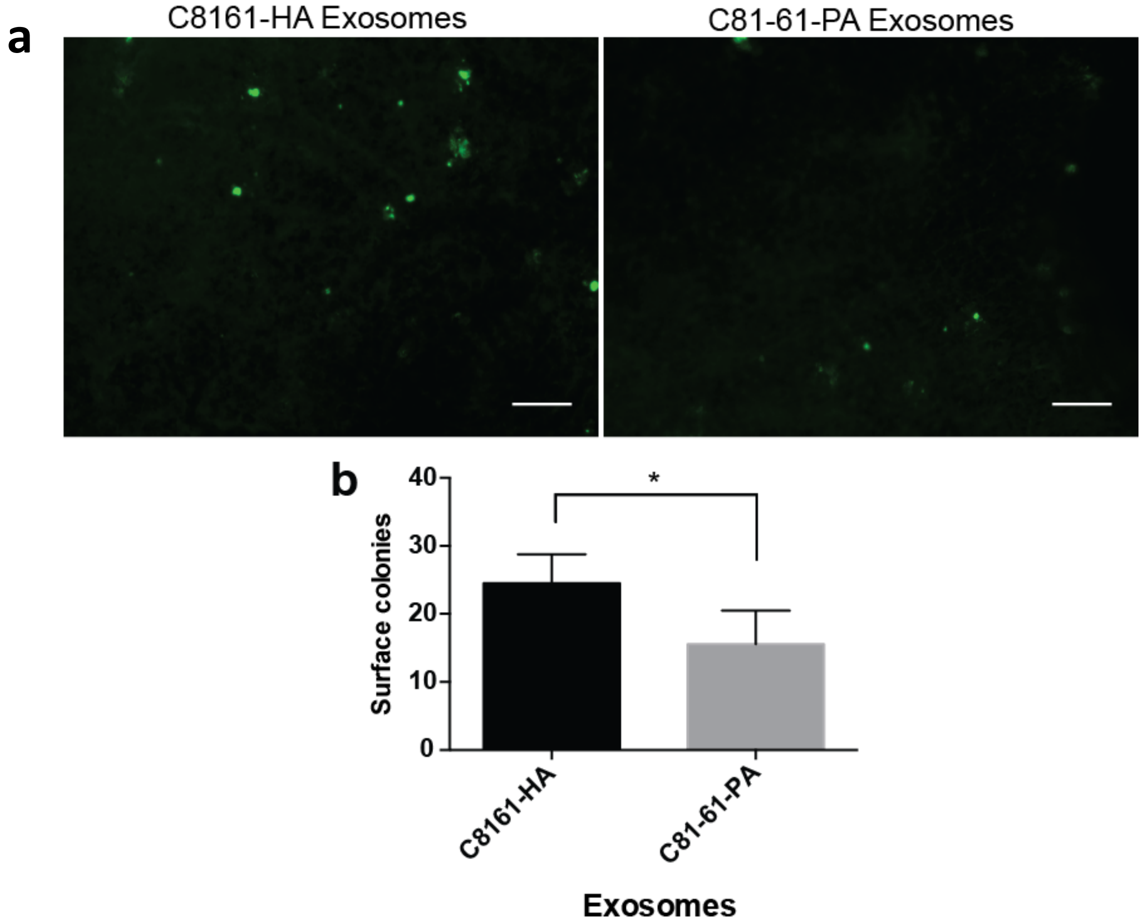

Supplementary Figure 4. Inhibition of lung extravasation by exosomes from naturally occurring metastatic and non-metastatic melanoma cells. Exosomes were isolated from poorly aggressive C81-61-PA and highly aggressive C8161-HA human melanoma cells (isogenic lines, a gift from Dr. M Hendrix). Nude mice were injected with the indicated exosomes three times 2 days apart, followed by injection of C81-61-PA cells (10^6) via the tail vein. The lungs colonies were assessed 24 hours post-injection. (**a**) Representative lung images. (**b**) Quantification of the surface colonies in the lungs of mice analyzed by fluorescence microscopy in (n=4 animals per group, a minimum of 5 fields per animal). * indicates $P < 0.05$, assessed by two-tailed Student's *t*-test. Means and s.d. values are shown.

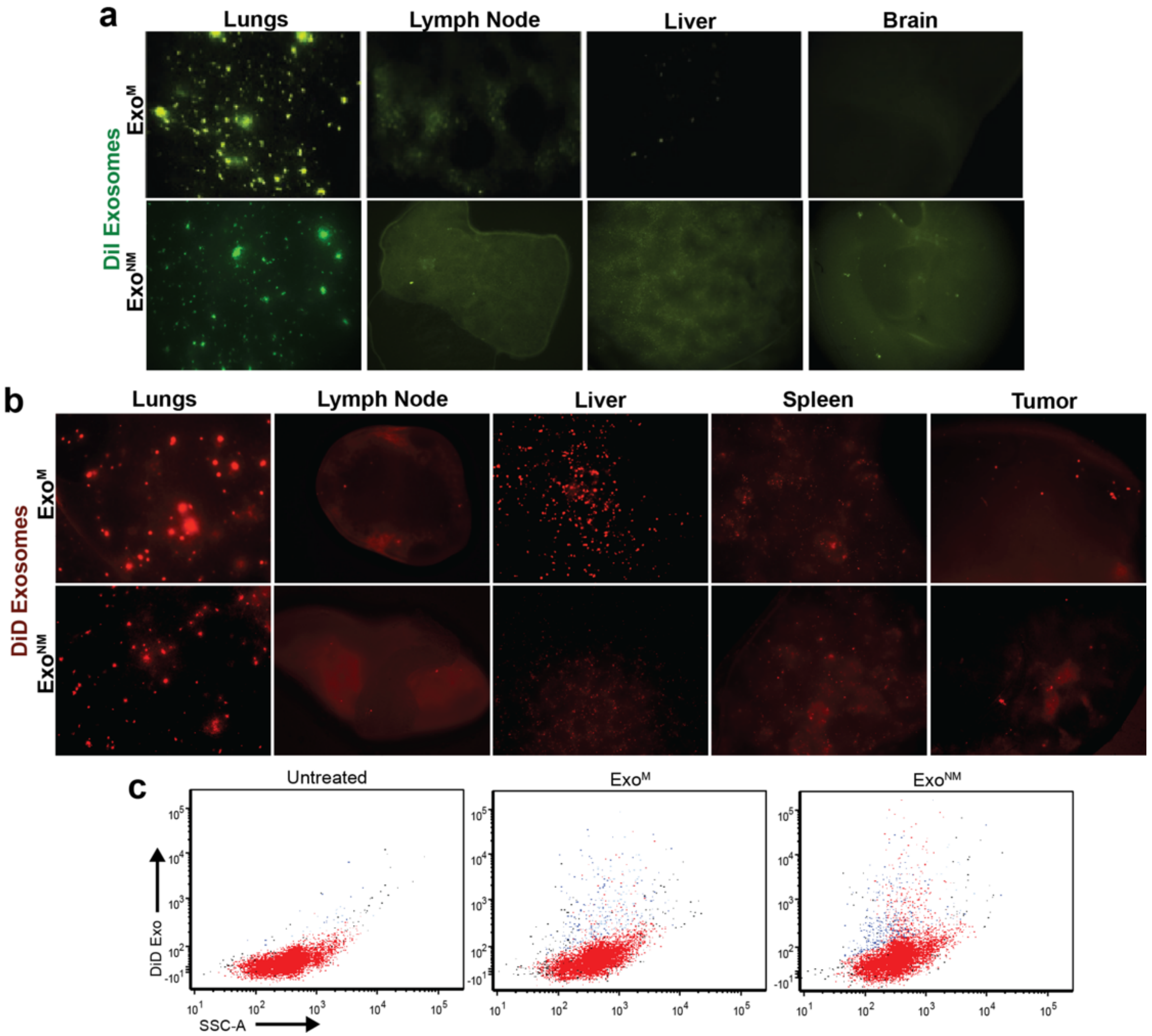

Supplementary Figure 5. Biodistribution of melanoma exosomes. Mice received tail vein injections of 10 µg melanoma exosomes (Exo^{M} and Exo^{NM}, as indicated) labeled with the fluorescent lipophilic dye DiI (green) or DiD (red). The biodistribution of melanoma exosomes were analyzed in tumor-free mice (**a**) tumor-bearing mice (**b**) and in bone marrow isolates (**c**).

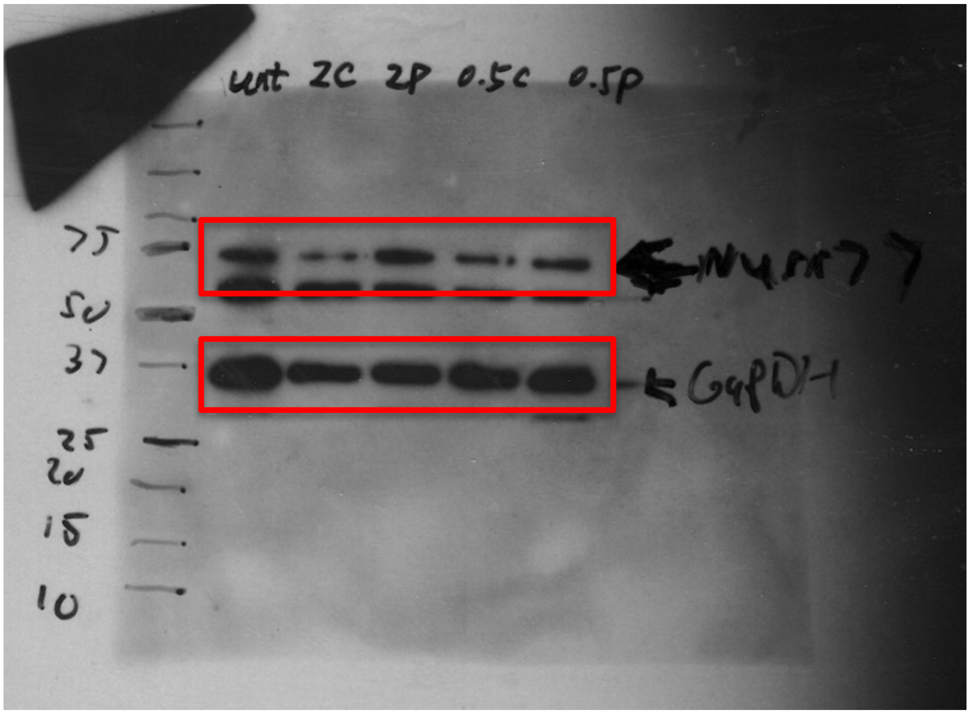

Supplementary Figure 6. Full-size Western blot analysis of Nr4a1 expresssion in primary mouse macrophages after treatment with Exo^{NM} and Exo^{M}. Western blot shows an increase in Nr4a1 protein in mouse BM monocytes treated with Exo^{NM} compared to Exo^{M} or baseline control. Red box indicates what is shown in ***Fig. 2d***.

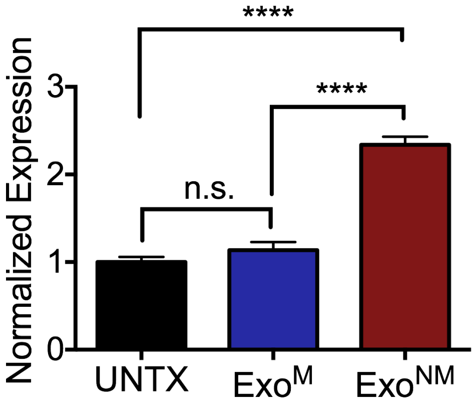

Supplementary Figure 7. Characterization of PMo markers in cultured cells. THP-1 cells were treated with Exo^{M} and Exo^{NM} (1μg/ml), where indicated. ITGB2 mRNA levels were evaluatedby q-RT-PCR (with β-actin as internal control) and normalized to untreated control, Untx. Three biological and two technical replicates were evaluated. **** indicates *P<0.0001* in pairwise comparison, using two-sided by Students' t-test. Means and s.d. values are shown.

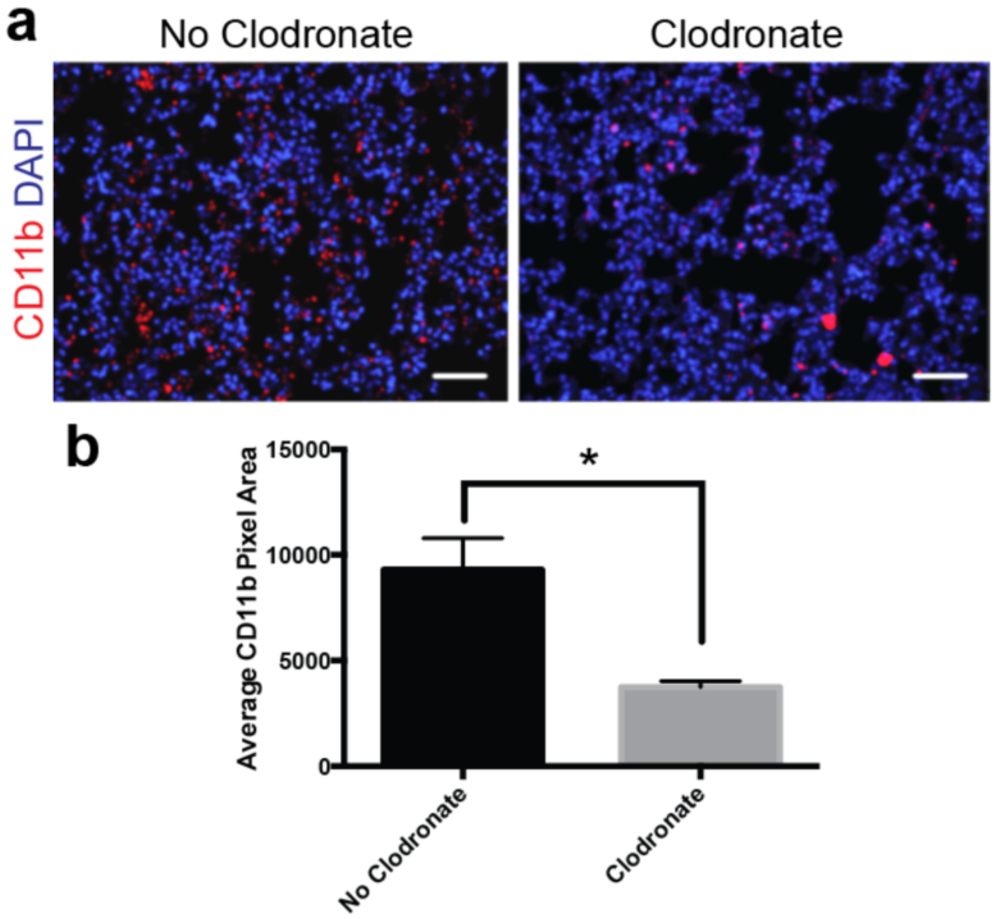

Supplementary Figure 8. Depletion of CD11b-positive cells with clodronate liposomes. C57BL/6 mice were treated twice with clodronate or control liposomes 96 and 48 hours prior to lung dissection. To confirm depletion, lungs were fixed, paraffin-embedded, sectioned and stained for CD11b. (n=3 animals per group, a minimum of 5 fields per animal). **(a)** Representative lung images. Scale bar: 50 µm. **(b)** quantitative analysis of the images using Elements software (Nikon). * indicates *P<0.05* calculated by two-sided Student’s *t*-test. Means and s.e.m. values are shown.

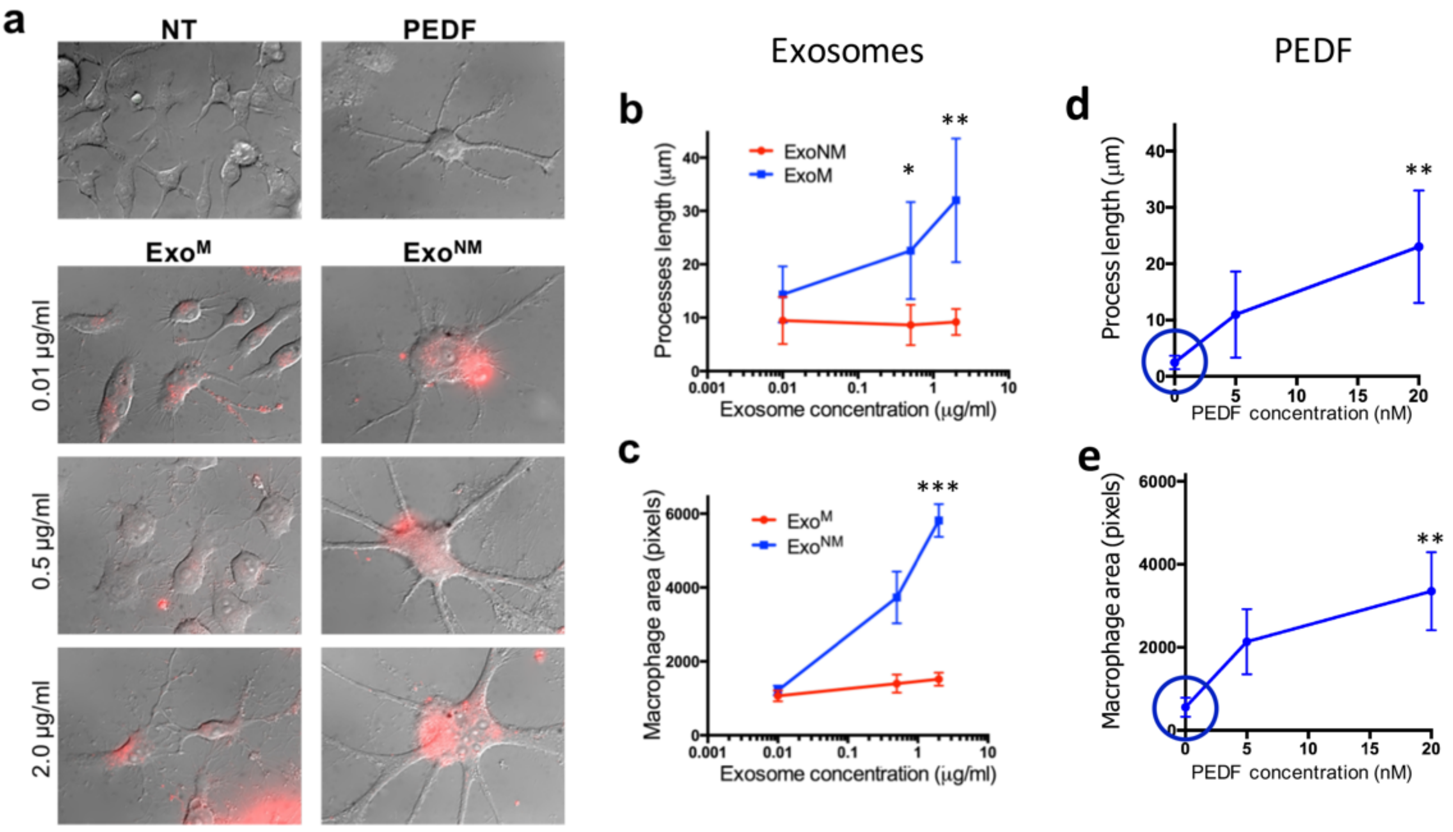

Supplementary Figure 9. Exo^{NM} cause the differentiation of RAW 264.7 macrophages. (**a**) RAW 264.7 macrophages were treated with 0.01, 0.5, and 2.0 µg/ml of DiI-labeled exosomes as indicated The controls included PEDF (rPEDF, 20 nM) or no treatment (NT). After 24 hours, the cells were analyzed by bright-field and fluorescence microscopy. DIC and fluorescence images are superimposed. The process length **(b)** and the macrophage surface area **(c)** were quantified to assess differentiation using ImageJ software (National Institutes of Health, Bethesda, MD). **(d, e)** process length and surface area in RAW macrophages treated with rPEDF. Note that plots in ***b*** and ***c*** are on a logarithmic scale and do not include baseline measurements (zero concentration), which are included in plots for rPEDF ***d*** and ***e***. Measurements were performed at 100x magnification of 20-30 randomly picked cells per data point. *, *P<0.05*; **, *P<0.01*; ***, *P<0.002* by Tukey's multiple comparison test, Bopferroni post-test. Means and s.d. values are shown.

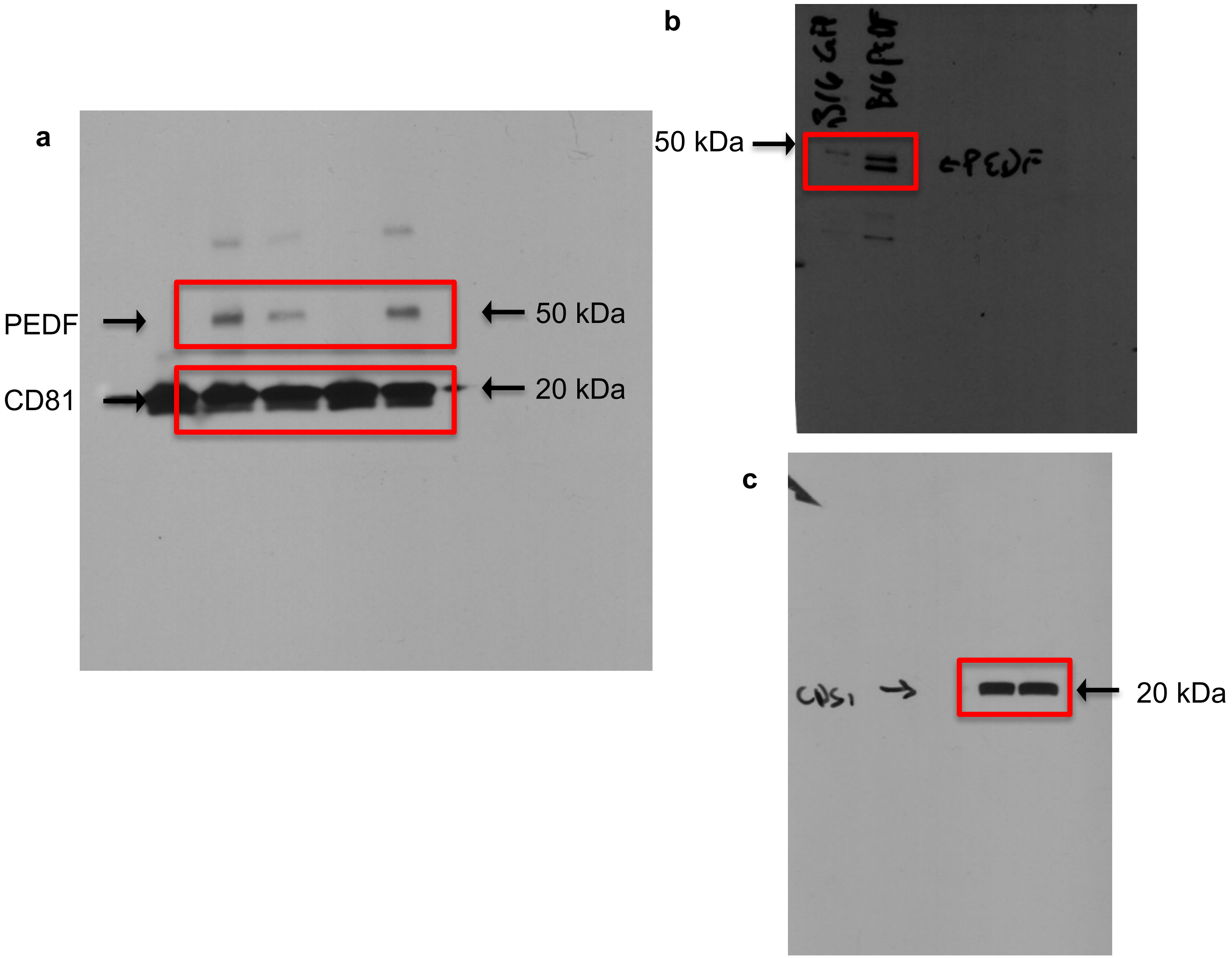

Supplementary Figure 10. Full-size Western blots of Exo^{M} andExo^{NM} presented in Fig. 5a. Exosomal protein was loaded at 10 μg per well. **(a)** PEDF enrichment in exosomes from non-metastatic melanoma cell lines (A375 control, A375 PEDF, SBcl2, C8161 HA, C81-61 PA). **(b)** PEDF enrichment. **(c)** CD81, a loading control for (b). **(d)** The correlation between cytoplasmi and exosomal PEDF levels in A375 cells. Red outline indicates panels presented in **Fig. 5a**.

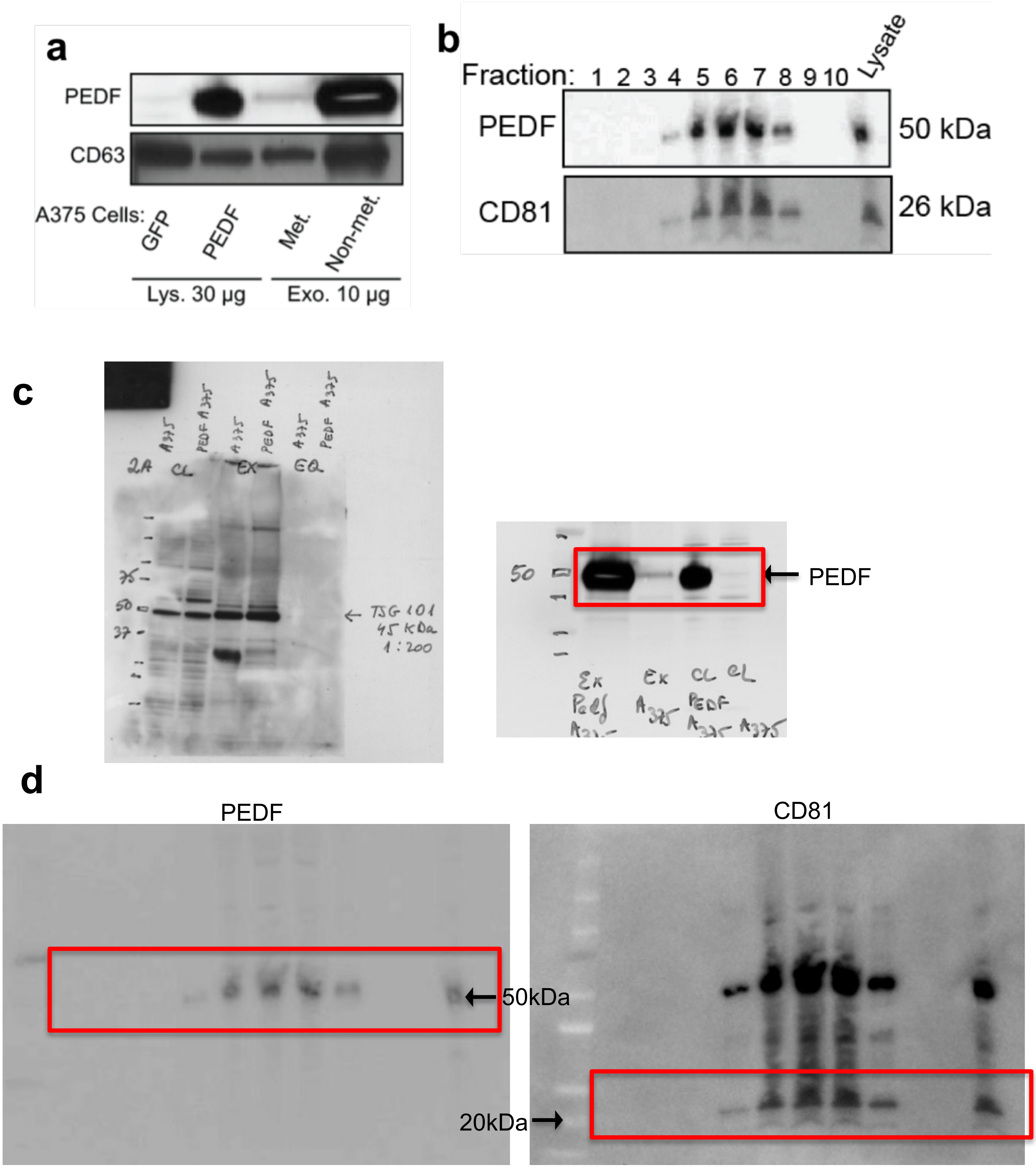

Supplementary Figure 11. Characterization of exosomal PEDF. (a) comparison of PEDF in cell lysates and in exosomes from the same cell lines. **(b)** Exosomes were isolated by gradient centrifugation and PEDF assessed in the fractions. Note that exosome-positive (CD81-containing) fractions also contain PEDF. **(c, d)** full-size blots used for ***a*** and ***b*,** respectively.

a

b

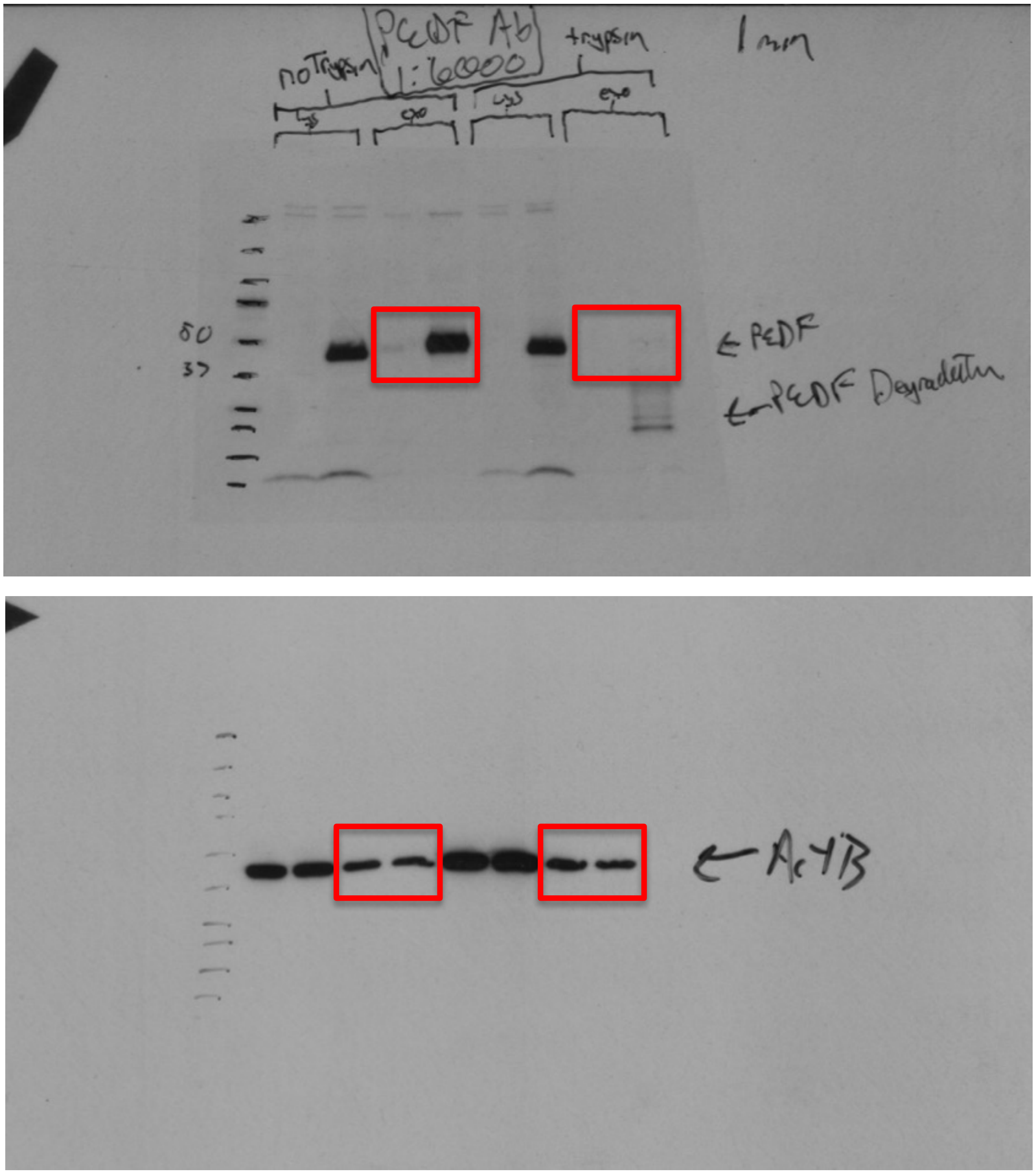

Supplementary Figure 12. Full-size source blots used in Figure 5b. (a) Cell lysates and exosomes were left intact or subjected to limited trypsin digestion. The blot is probed with PEDF antibody. **(b)** The same blot re-probed for beta actin. Red rectangels indicate areas presented in ***Figure 5b***. Cell lysates and exosomal protein were loaded at 30 and 10 μg per well, respectively.

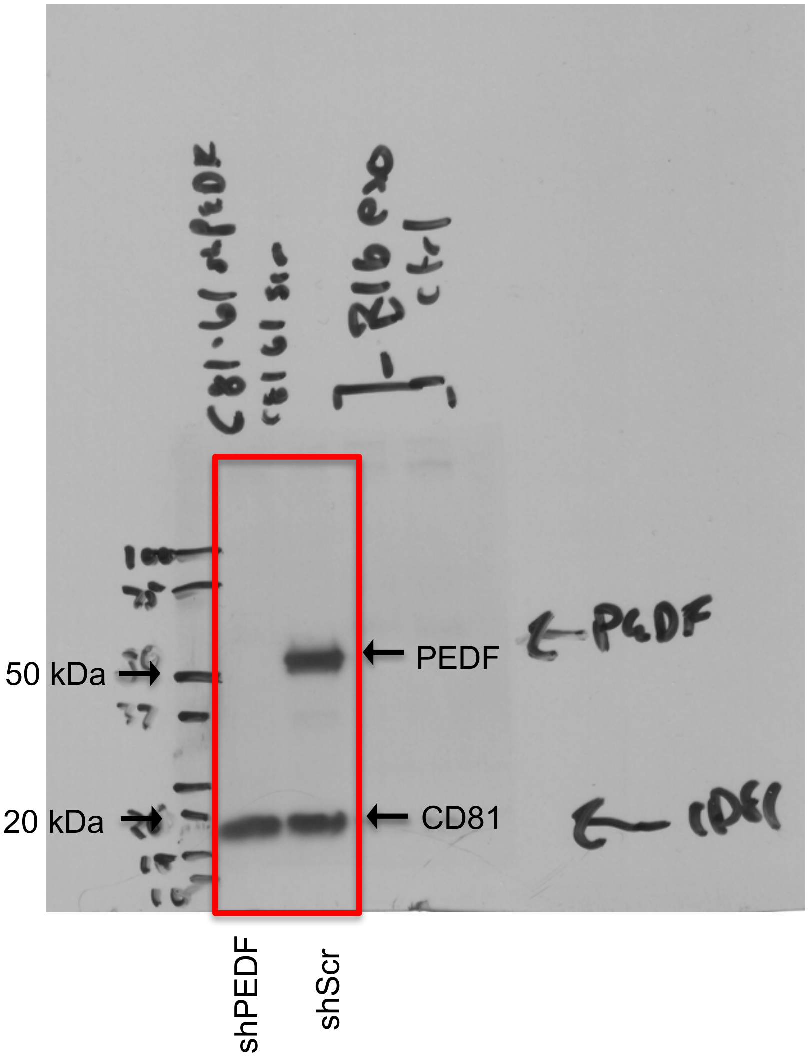

Supplementary Figure 13. Knockdown of PEDF in non-metastatic C81-61 PA cells. PEDF, which is endogenously expressed in tis cell line was knocked down from C81-61PA cells using shRNA. Western blotting shows loss of PEDF in exosomes isolated from C81-61 PA shPEDF cells transfected with letiviral shRNAmiR VL2HS_221662 from Open Biosystems (Huntsville, AL, USA). Non-silencing shRNAmir sequence (shScr) cloned into pGIPz vector has with no homology to known mammalian genes (Open Biosystems). These cells have been generated and published by our lab previously[2]; here we confirm the presence or the lack of PEDF in the respective exosomal preparations. CD81 is used as a loading control. The relevant portion of the blot is marked with red outline.

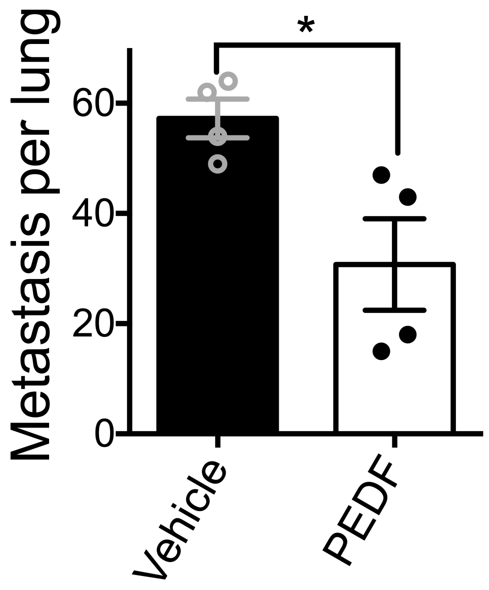

Supplementary Figure 14. Treatment with short PEDF peptide mimetic attenuates lung metastasis by B16F10 melanoma. C57Bl6 mice in groups of 4 were inoculated with B16F10 melanoma cells (10^6 cells per animal) and treated with daily i.p. injections of PEDF short peptide mimetic that reproduces anti-cancer and anti-metastatic activity of PEDF through its 34-mer surface epitope[3] (60 mg/kg^{-1}) or with equal volume of control vehicle (PBS). Note reduced metastasis in peptide-treated group compared to vehicle control. * indicates $P<0.05$ as calculated by two-tailed Student’s t-test.

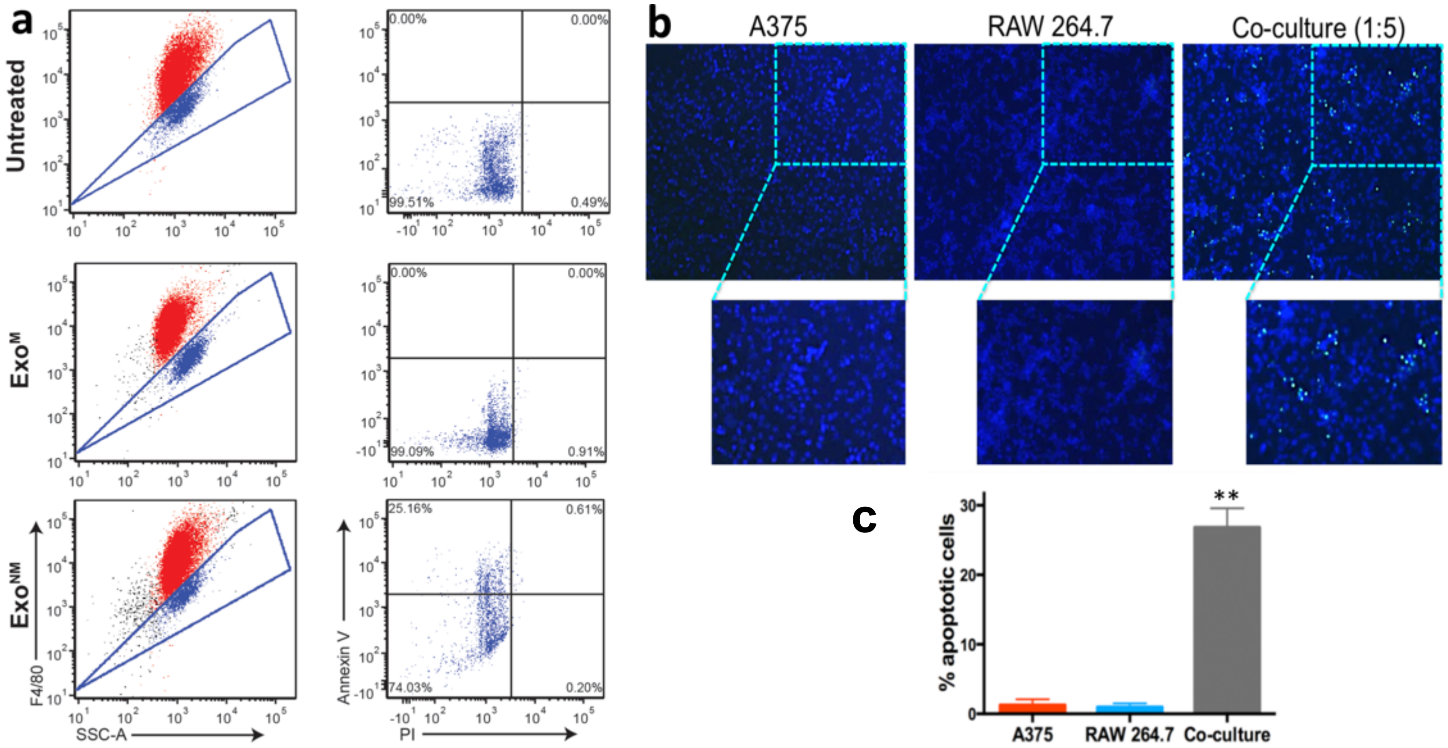

Supplementary Figure 15. In co-cultures with macrophages, Exo^{NM}-dependent apoptosis is limited to melanoma cells. RAW 264.7 macrophages were grown in co-culture with A375 melanoma cells at a 5:1 ratio and treated with either Exo^{M} or Exo^{NM} (3 µg/ml for 24 hours). Cells were stained with a fluorophore-tagged F4/80 antibody to detect macrophages and apoptotic cells detected with annexin V for FACS (**a**) or by in situ TUNEL for evaluation by fluorescence microscopy coupled with Nikon Elements software (**b, c**). **(b)** Representative 10x images and close-ups of Tunel staining (green, TUNEL; blue, DAPI). **(c)** Quantitative evaluation of TUNEL staining. All conditions were evaluated in biological triplicates and a minimum of 5 random fields evaluated per condition. ** indicates *P<0.01* calucalted by two-sided Student’s t-test compated to A375 monoculture. Mean and s.e.m. values are shown.

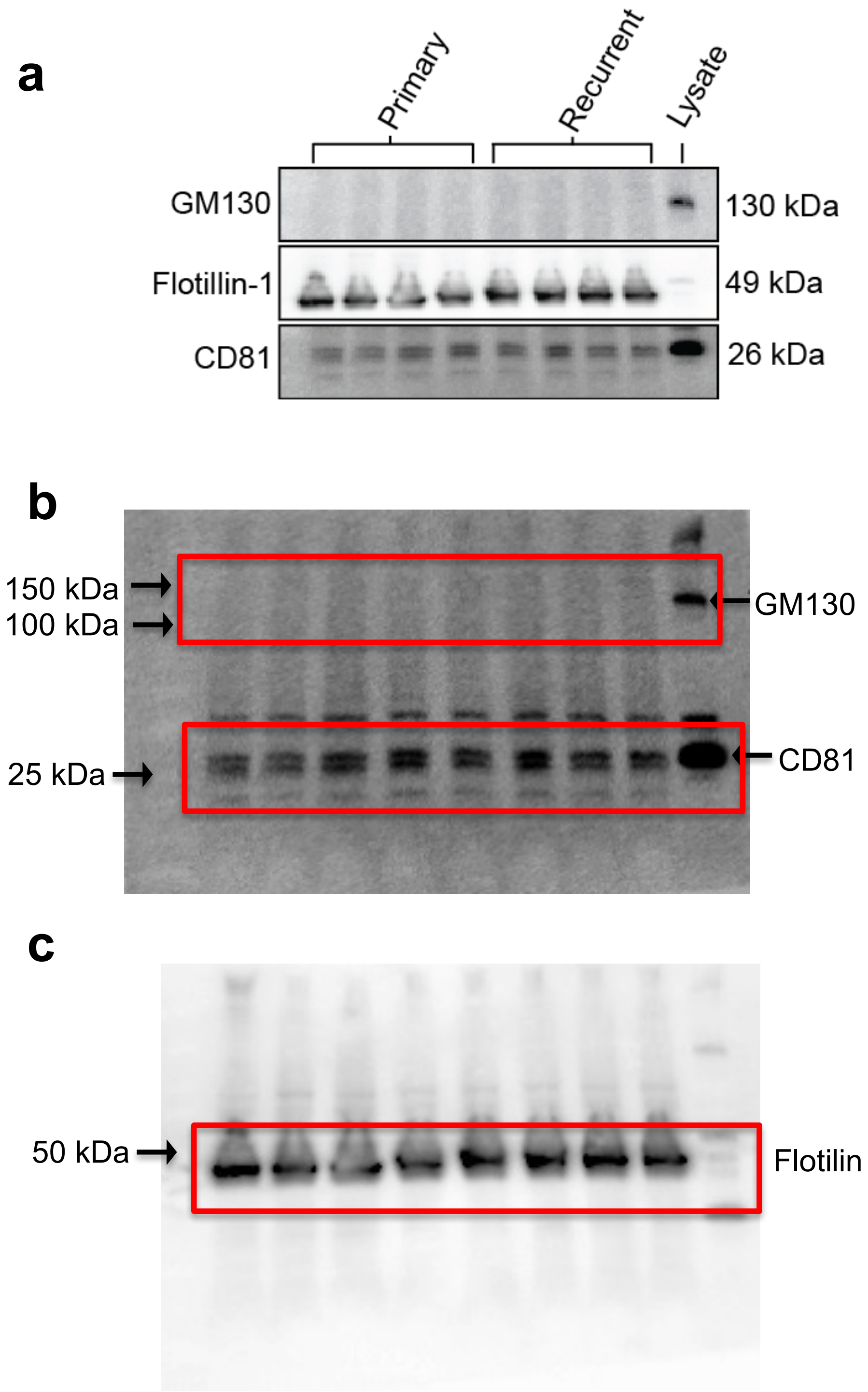

Supplementary Figure 16. Characterization of exosomes from the sera of melanoma patients. **(a)** Exosomes were isolated from the sera of melanoma patients with recurrent metastatic disease and disease that did not spread from the primary site. Preparation was analyzed for the presence of exosome markers CD81 and Flotillin-1. In contrast, exosome patient samples did not contain the golgi marker GM130, which was present in the lysate of A375 melanoma cells. **(b, c)** Full-size source blots for panels in ***a*.**

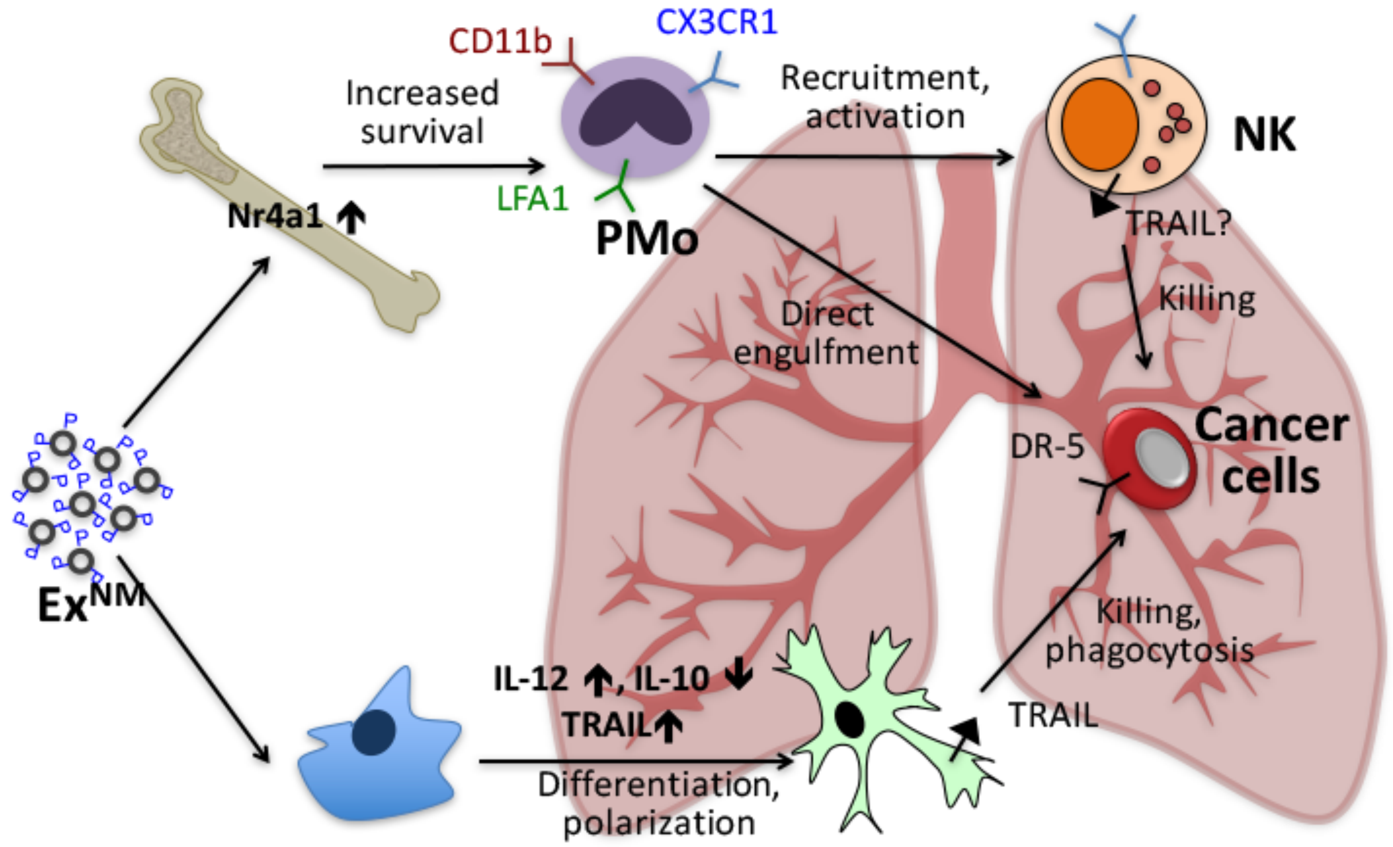

Supplementary Figure 17. Schematic representation of immune surveillance events triggered by non-metastatic exosomes. Non-metastatic exosomes **(**Exo^{NM}) home to the bone marrow and to the lung. In the bone marrow, they home to CD11b positive myeloid cells and increase the expression of NR4A1 receptor, thus increasing survival and overall numbers of patrolling monocytes (PMo), which eradicate cancer cells through recruitment of the natural killer (NK) cells and by direct engulfment of the cancer cells. In the lung, Exo^{NM} home to macrophages and cause macrophage differentiation, manifested by decreased expression of M2 marker, IL-10, and increased production of M1 marker, IL-12. In addition, macrophages undergo profound morphological changes, with multiple dendrite-like processes, and increased expression of death ligand TRAIL, leading to the melanoma cell killing and phagocytosis. These events are dependent on PEDF, which is displayed on the exosomes outer surface.

Supplementary Table 1:
Human serum samplestested in extravasation assay

Patient Type	ID	Age at Diagnosis	Gender	Histological Type	Thickness (mm)	Ulceration	Mitotic index	Disease Stage	Tumor stage	PEDF, ng/µg exosomal protein
Primary	07-004	68.9	F	Superficial spreading melanoma	0.45	Absent	NONE	Stage I	T1A	0.053323
Primary	07-079	56.1	M	Nodular melanoma	5.5	Present	MANY	Stage II	T4B	0.096080
Primary	07-243	28.4	F	Superficial spreading melanoma	0.35	Absent	NONE	Stage I	T1A	0.012370
Recurrent	06-042	44.3	F	Nodular melanoma	2.8	Absent	MANY	Stage III	T3A	0.053022
Recurrent	07-127	69.5	F	Superficial spreading melanoma	1.75	Absent	MODE RATE	Stage IV	T2A	0.239748
Recurrent	07-250	59.6	M	Superficial spreading melanoma	1.1	Absent	NONE	Stage IV	T2A	0.189861
Tumor-free	C10106	N/A	M	N/A	N/A	N/A	N/A	N/A	N/A	0.005209
Tumor-free	C11065	N/A	F	N/A	N/A	N/A	N/A	N/A	N/A	0.013202
Tumor-free	C10125	N/A	F	N/A	N/A	N/A	N/A	N/A	N/A	0.034803

Supplementary Table 2:
Primary and secondary antibodies used in the study

**Primary**				
**Protein**	Clone	Use	Dilution	Manufacturer
**Beta-Actin**	13E5	WB	1:5000	Cell Signaling Tech.
**CD11b**	M1/70	FC	1:100	BD Pharmingen
**CD45**	30-f11	FC	1:100	Biolegend
**CD63**	TS63	WB	1:1000	Abcam
**CD81**	B-11	WB	1:500	Santa Cruz Biotech.
**CX3CR1**	Polyclonal	IHC, FC	1:100, 1:200	Novus, Biolegend
**F4/80**	BM8	IHC	1:400	eBiosciences
**Gr1**	Rb6-865	FC	1:200	BD Pharmingen
**NK1.1**	PK136	FC	1:100	Biolegend
**Nurr77**	EPR3209	WB	1:1000	Abcam
**PEDF,**	Polyclonal	WB, Blocking	1:2000, 1:500	Bioproducts MD
**TSG101**	4A10	WB	1:2000	Abcam
**Ly6C**	HK1.4	FC	1:200	Biolegend
**GM130**	35/GM130	WB	1:1000	BD Transductions
**TBP**	51841	WB	1:1000	Abcam
**Secondary**				
**Anti-Rabbit IgG**	Polyclonal	WB secondary-HRP	1:5000	Jackson Immunoresearch
**Anti-Mouse IgG**	Polyclonal	WB secondary-HRP	1:5000	Jackson Immunoresearch
**Anti-Rat IgG**	Polyclonal	IHC secondary-Cy5	1:100	Jackson Immunoresearch
**Anti-Rabbit IgG**	Polyclonal	IHC secondary-488	1:100	Jackson Immunoresearch

Supplementary Table 3:
PCR primes used in the study

Gene	Primer
**Actb**	Fwd: 5'-aagtcagtgtacaggtaagcc-3'
	Rev: 5'-gtcccccaacttgagatgtatg-3'
**CX3CR1**	Fwd: 5'-gaacacagtcccaaagacca-3'
	Rev: 5'-tgtcctcggaacaccaca-3'
**IL10**	Fwd: 5'-ctccaagaccaaggtgtctac-3'
	Rev: 5'-ggagtccagcagactcaatac-3'
**IL12**	Fwd: 5'-gggagaagcagacccttaca-3'
	Rev: 5'-ttcaggcggagctcagatag-3'
**ITGB2**	Fwd: 5'-tcactccattgctgcagaag-3'
	Rev: 5'-gggagctgtctgaggact-3'
**Nr4a1**	Fwd: 5'-cttcaggctgtctgttcgg-3'
	Rev: 5'-cgccaagtacatctgcct-3'
**Tnfsf10**	Fwd: 5'-cagccctaaagtacccagtaatc-3'
	Rev: 5'-cacatctgtcctgaggtttctac-3'

1 Orgaz, J. L. *et al.* 'Loss of pigment epithelium-derived factor enables migration, invasion and metastatic spread of human melanoma'. *Oncogene* **28**, 4147-4161, doi:10.1038/onc.2009.284 (2009).

2 Mirochnik, Y. *et al.* Short pigment epithelial-derived factor-derived peptide inhibits angiogenesis and tumor growth. *Clin Cancer Res* **15**, 1655-1663, doi:10.1158/1078-0432.CCR-08-2113 (2009).
