# Supplementary material for: Pre-metastatic cancer exosomes induce immune surveillance by patrolling monocytes at the metastatic niche
Source: Nat Commun. 2017 Nov 6;8:1319. doi: 10.1038/s41467-017-01433-3 (PMC5673063; doi:10.1038/s41467-017-01433-3)
Supplement: Supplementary file 3 — Description of Additional Supplementary Files [file 41467_2017_1433_MOESM3_ESM.pdf]

Description of Additional Supplementary Files

File Name: Supplementary Movie 1
Description: The effect of ExoM on macrophage differentiation. RAW 264.7 macrophages were plated in glass bottom plates and treated with 3 µg/ml of ExoM. Images were taken every 15 minutes for 16 hours focusing on the formation of dendritelike processes.

File Name: Supplementary Movie 2
Description: The effect of ExoNM on macrophage differentiation. Time-lapse images of RAW264.7 macrophages treated with 3 µg/ml of ExoNM. Images were taken every 15 minutes for 16 hours focusing on the formation of dendrite-like processes.
