# Supplementary material for: Pre-metastatic cancer exosomes induce immune surveillance by patrolling monocytes at the metastatic niche
Source: Nat Commun. 2017 Nov 6;8:1319. doi: 10.1038/s41467-017-01433-3 (PMC5673063; doi:10.1038/s41467-017-01433-3)
Supplement: Supplementary file 2 — Peer Review File [file 41467_2017_1433_MOESM2_ESM.pdf]

Reviewers' comments:

Reviewer #1 (Remarks to the Author):

The article illustrates the role of exosomes released by non-metastatic cancer cells in immune surveillance. The article builds on some previous published works on pre-metastatic niche and immune surveillance either in the context of cells and exosomes. The manuscript in its current form contains too many big claims that are not supported by data, severely flawed and is hence not interesting/exciting. The quantity or the quality of work seems not be of Nature Communications level.

1) The physiological relevance of this study is questionable and the central concept of the study is severely flawed. As all primary tumours start as non-metastatic, if the observations recorded here are true, there will be no metastasis for any cancer. This is clearly not the case. Even if the authors argue that non-recurring non-metastatic primary melanoma patients have exosomes that blocks metastasis, the authors do not seem to have a hypothesis and data to support this.
2) How did the authors separate A375 metastatic and non-metastatic? It is unclear here.
3) The data on PEDF is not convincing. Can the authors knockdown/knockout PEDF from the cells and show similar effect? Also, can the authors over express PEDF in metastatic cells.
4) The article is poor on exosomes isolation and characterization. The authors have used commercial kits for isolation (plasma) and it is well established (multiple articles and conference presentations) in the field that the commercial kits are quick and dirty. They tend to precipitate non EV material and hence confound the whole study and data interpretation (pubmed: 25317274). See the position statement by the International Society of Extracellular Vesicles (pubmed: 25536934) RAISING ALARM OVER THIS ISSUE!
5) The article is poor in exosomes characterization from plasma. Again, see the position statement by the International Society of Extracellular Vesicles (pubmed: 25536934) on the minimal requirements of publication in EVs.
6) Can the authors provide data with exosomes depleted plasma? This control is lacking. Also, what about the metastatic melanoma plasma exosomes as a control?

Reviewer #2 (Remarks to the Author):

The manuscript by Plebanek et al. addresses the role of exosomes from nonmetastatic melanoma cells and whether they may inhibit metastasis. This is an interesting idea and the flipside of recent studies showing that exosomes from metastatic melanoma cells promote metastasis by seeding premetastatic niches. The authors compare exosomes purified from metastatic and nonmetastatic isogenic melanoma cells and conclude that nonmetastatic melanoma exosomes induce an immune response in the form of patrolling monocytes. The exosomes go to the bone marrow to induce formation of the patrolling monocytes, which then circulate throughout the body and quickly eliminate extravasating/extravasated tumor cells in the lung. This is a mechanism by which exosomes may have an anti-tumorigenic activity by activating the innate immune system to eliminate cancers cells and thereby limit metastasis. For a molecular mechanism, the authors provide evidence that PEDF on NM-exosomes plays an important role in inducing macrophage differentiation and downstream cancer cell killing. They also isolate exosomes from patient samples and report that exosomes from primary melanomas inhibit metastasis in a xenograft model whereas recurrent melanoma-derived exosomes enhance metastasis, compared to healthy controls. There are a number of nice experiments in this manuscript and it is generally well-written. However, there are a number of issues that should be addressed before publication. A major issue is that in many experiments, there is no control that did not receive exosomes. The comparison in those cases is only to metastatic exosomes, so the difficulty is in concluding whether the NM-Exo are inducing an effect or whether the M-Exo are instead the exosomes with

the major biologic activity, since it is in fact a relative comparison. This is important, since the major novel finding is that nonmetastatic exosomes in fact limit metastasis (as opposed to not promoting it, which is already reported many times by the Lyden group). I think in the end the authors will be “right”, but this control and a few others listed below are really important.

Major Points
1. The mouse experiments in Fig 1 should have an untreated or diluent treated control, as it is unclear whether the NM-exosomes are inhibiting metastasis or just don’t have the promoting effect that M-exosomes do.
2. Likewise, Figs 4 and 5 need untreated controls.
3. Fig 2d, the Western blot of Nr4a1 is not convincing. The antibody specificity should be checked, e.g. by a knockdown or IP with a separate antibody. Also, multiple independent reps should be quantitated.
4. The results in Fig 3c (images) should be quantitated across multiple images and independent reps. Likewise for Fig 4d,e
5. Supp Fig 5 needs better images and quantitation from multiple images.
6. Supp Fig 6 needs untreated or diluent treated control.
7. Supp Fig 7 needs untreated or diluent treated control.
8. It is not so clear to me that the macrophage depletion experiments are as “all or nothing” in terms of mediating the effects of NM-Exo and the NK depletion experiments are “partial effect”. That is, the differences observed with immune cell depletion look similar for macrophage depletion and NK depletion although the statistics say otherwise. Some revisiting of the statistical analysis, the n value, and interpretation of the data may be warranted. It’s not that I think the authors are so off in their conclusions but it seems likely that with added data, the statistics might be similar.
9. Along these lines, the actual n value that goes into statistics for all quantitations should be stated in the figure legends, e.g. n=XX lung fields from XX mice for Fig 4c.
10. To really show that PEDF is responsible for the macrophage-promoting effects of NM-Exo, depletion of PEDF (e.g. by shRNA in the exosome-producing cells) from NM-Exo should be done and then the effect of equal numbers of PEDF-depleted Exo should be compared to control Exo from the same cell line. The neutralizing antibody is not good enough, because the antibody may just prevent the exosomes or other proteins on the exosomes from interacting with cells.
11. Fig 7, the statement that Peinado et al. found more exosomes in recurrent patients is not exactly true. To be precise, they reported finding no difference in exosome number in patients of any stage vs. normal, but did find more exosomal protein in Stage IV. I also don’t know how much can be said about exosomal protein when the exosomes were isolated by ExoQuick (Fig 7a).
12. The media used for collecting exosomes should be specified. “Exosome-free” media could mean many things.
13. At least some key experiments should be repeated using density gradient purified exosomes to show validation by an extra-pure vesicle population.
14. It would be good to have some non-exosome markers on the Western blots in Fig 1 to show purity of prep.

Minor Points
15. May want to do more referencing for role of immune cells in metastatic cell clearance, e.g. Malladi et al., Cell, 2016 for NK cells
16. Some reference for the isogenic C8161 cell lines should be given.

Reviewer #4 (Remarks to the Author):

Understanding the role of exosomes in tumor progression, and in particular at the pre-metastatic niche, is most warranted. The potential novelty of this work relates to the anti-tumor effect of exosomes that are secreted by non-metastatic melanomas, by inducing an immune surveillance at

the pre-metastatic niche. However, authors conclusions are not supported by the data shown, and further studies with important controls are required to substantiate the key conclusions offered by this study.

Major:
Figure 1: "This data demonstrated that “non-metastatic” exosomes block lung metastasis." This effect could be due to an enhancement of metastasis induced exosM (as already published by Peinado et al, Nat Med 2012). Before this conclusion can be drawn, the authors should provide a control which consists of administering A375 cells after conditioning the mice with a vehicle. Along the same lines, does the conditioning with exoNM inhibit basal metastasis of A375 cells?

Supplementary figure1: What is the difference in exosome secretion in terms of quantity of vesicles? How many particles 10ug refers to? It has been published that exosomes from metastatic melanoma cells are enriched in proteins. Therefore, the authors should refer to a quantity of exosomes and not to a protein quantity.

Supplementary Figure 4: According to the text, one expects to see a comparison of the biodistribution of exos NM and exosM in mice. Is this the case?

Figure 3: Staining of more specific markers of PMo than CX3CR1 should be performed in the lungs of mice pre-conditioned with ExoNM. Again, a control of mice treated with the vehicle has to be included.

Fig4e: Phagocytosis of fluorescent-melanoma cells should be quantified.

Figure 6a: In order to demonstrate that PEDF is enriched in exosomes, equal amounts of proteins of whole cell and exosome lysates should be analyzed by western blot in a panel of melanoma lines (metastatic or not) without manipulation of PEDF.

Figure 6d-g: The same experiments should be done with primary macrophages.

Supplementary figure 8: Which treatment is represented by these images? Where are the others conditions (non treated, exosNM and exosM treated)?

Figure 7: Showing PEDF level in exosomes isolated from the different groups of patients and using blocking antibody against PEDF and/or clodronate would improve the message of this study. What is the quantity of exosomes injected in each conditions?

Minor:
Sup1a and b: Font too small, not readable

Page 5: Tumor bearing mice is written twice (line 16).
Figure 2b: The authors state" in the total myeloid population, Ly6Chi subpopulations were similar upon treatment with control ExoM or with ExoNM. However, only ExoNM caused a significant increase of the Ly6Clow population".
Cells are either Ly6C high or low. If there is a decrease in Ly6C low subpopulation percentage, the LyC high subpopulation should also be affected. This is what is actually seen on the flow plots. This sentence has to be changed.
" In previous studies by our laboratory as well as other groups, it was established that pigment epithelium-derived factor (PEDF) renders melanoma cells non-metastatic. Importantly, PEDF is also expressed by most non-metastatic cell lines19" : Another reference is required.

Fig. 6f, h: There is no panel h but a g.

We thank the reviewers for their constructive criticisms and truly appreciate their careful attention to the masnucript. We believe that in addressing the reviewers' concerns we have significantly improved the analysis and substantiated the claims of the study. Especially, the main concern by all three reviewers , the lack of baseline control for the *in vivo* studies was fully justified. In all key *in vivo* experiment it has been added and $Ex^{NM}$ treatment invariably falls below the baseline, indicating that the original claims are likely fair. We did our best to address other reviewers' concerns, all of them productive and fair. Below please find point-by-point response to the reviewers' comments. All changes in the manuscript are highlighted in gray.

**Reviewer 1.**

**1)** The physiological relevance of the study is questionable. As all primary tumours start as non-metastatic, if the observations recorded here are true, there will be no metastasis for any cancer. This is clearly not the case.

- We respectfully disagree with the reviewer. Our study, in line with findings by others, indicates, that at least in melanoma, there is a stage in progression where tumor cells are capable of dissemination but their metastatic spread is curtailed by immune surveillance. This is in agreement with existing views on cancer progression. The ability to suppress host surveillance is gained in the course of tumor progression in multiple ways, e.g. increased expression of PD-L1. This is a complex process, in which exosomes apparently play a part, as is suggested by our study.

**2)** Even if the authors argue that non-recurring non-metastatic primary melanoma patients have exosomes that blocks metastasis, the authors do not seem to have a hypothesis and data to support this.

- Our data shows that the exosomes collected from the sera of non-recurrent patietns suppress extravastion of melanoma cells *in vivo*, compared to the exosomes from healthy volunteers or exosome-depleted sera. Consistent with findings by others, exosomes from metastatic patients increase the number of extravasated cells (now **Fig. 6**). These results additionally support our hypothesis. To match these results using cell-derived exosomes, we have incorporated lung colonization and extravasation experiments using mouse melanoma cells (B16F10) and their exosomes, with additional control (no exosomes injected, Untx), providing the baseline for metastasis in the absence of exosome influence (**Fig. 1** and thereafter). In all the experiments, non-metastatic exosome condition is significantly different (lower) from vehicle control, while metastatic exosomes increase baseline metastasis. The supporting experiments using human cell lines have been relegated to Supplementary Materials.

**3)** How did the authors separate A375 metastatic and non-metastatic? It is unclear here.

- We apologize for the confusing statement. Non-metastatic A375 and B16F10 cells were generated in our previous studies by overexpression of Pigment Epithelium Derived Factor (Orgaz et al., Oncogene. 2009;28(47) :4147-61) or selected by the Hendrix group with respect to their ability to form spontaneous metastasis from subcutaneous tumors (Yohem et al., Cancer Lett. 1989 May;45(2):135-43). This is now stated in the text, with appropriate references provided for all cell lines (**p. 4, p. 5**).

**3)** The data on PEDF is not convincing. Can the authors knockdown/knockout PEDF from the cells and show similar effect? Also, can the authors over express PEDF in metastatic cells.

- Please see response to comment 2 and response to Reviewer 2 comment 10. We also add evaluation of exosomal PEDF content in human serum exosomes where we show that PEDF levels correlate with survival (**Fig. 6d**).

**4)** The article is poor on exosomes isolation and characterization. The authors have used commercial kits and it is well established that the commercial kits are quick and dirty. They tend to precipitate non EV material and hence confound the whole study and data interpretation. See the position statement by the International Society of Extracellular Vesicles (pubmed: 25536934).

- We apologize for the confusion caused by careless proofreading of Methods, which resulted in misunderstanding. With cell lines as a source, exosomes were always isolated by differential ultracentrifugation/ sucrose gradient, as is now stated in Methods section (**p. 14**). The quality of preparations was ascertained by TEM, dynamic light scattering and nanotracking as well as analysis of common exosome markers and negative markers. Ultracentrifugation is used in multiple studies by the leading groups in the field (Hoshino et al., Tumour exosome integrins determine organotropic metastasis. Nature. 2015 Nov 19;527(7578):329-35. PMID: 26524530; Melo et al., Glypican-1 identifies cancer exosomes and detects early pancreatic cancer. Nature. 2015 Jul 9;523(7559):177-82.. PMID: 26106858). To further improve on isolation/purification we incorporated an experiment where we demonstrate PEDF presence in exosomal fractions after gradient centrifugation.

**5)** The article is poor in exosomes characterization from plasma. See the position statement by the International Society of Extracellular Vesicles (pubmed: 25536934) on the minimal requirements of publication in EVs.

- We were unfortunately limited by the use of archival samples, which were in limited supply but key to verification of conclusions from tissue culture studies. We were therefore confined to more economical isolation techniques. ExoQuick precipitation was followed by affinity purification using CD63 antibody column (Qiagen) to

eliminate non-exosomal material (**p. 14-15**). To ascertain the quality of serum exosomes we have added characterization by TEM (**Fig. 6A**) and Western blot (**Supplementary Fig. 10**). Since exosomes obtained by ultracentrifugation and by this two-step protocol yielded similar results, we believe thse results to be accurate.
**6)** Can the authors provide data with exosome-depleted plasma? This control is lacking. Also, what about the metastatic melanoma plasma exosomes as a control?
- In response the reviewers' request we have included exosome-depleted plasma (baseline), which does not alter the extravasation compared to exosomes form healthy volunteers. The metastatic melanoma exosomes were included previously (marked as **Recurrent**) (**Fig. 6b, c**).

**Reviewer #2**:
**General comment:** In many experiments, there is no control that did not receive exosomes. The comparison in those cases is only to metastatic exosomes, so the difficulty is in concluding whether the NM-Exo are inducing an effect or whether the M-Exo are instead the exosomes with the major biologic activity, since it is in fact a relative comparison. This is important, since the major novel finding is that nonmetastatic exosomes in fact limit metastasis (as opposed to not promoting it, which is already reported many times by the Lyden group). I think in the end the authors will be right but this control and a few others listed below are really important.
- We agree with the reviewer regarding the importance of the baseline control and have performed the key lung colonization and extravasation experiments using mouse B16F10 melanoma cells and their exosomes in immune competent mice, and additional control with no exosomes injected (vehicle), now included in all relevant figures (**Fig. 1 trhough 4**). In all the experiments, non-metastatic exosomes are significantly different from vehicle control. The experiments using human cell lines and exosomes have been now relegated to Supplementary Materials

**Other Major Points:**
**1)** The mouse experiments in Fig 1 should have an untreated or diluent treated control, it is unclear whether the NM-exosomes are inhibiting metastasis or just don't have the promoting effect of M-exosomes.
- Provided as requested

**2)** Likewise, Figs 4 and 5 need untreated controls.
- Provided as requested

**3)** Fig 2d, the Western blot of Nr4a1 is not convincing. The antibody specificity should be checked, e.g. by a knockdown or IP with a separate antibody. Also, multiple independent reps should be quantitated.
- We have included Western blot with different antibody, with similar results. Densitometry analysis shows a clear dose response to Exo$^{NM}$ (**Fig. 2d**). We have also added Nr4a1 staining of the lung tissue with quantitative analysis, showing similar changes (**Fig. 2g, h**).

**4)** The results in Fig 3c should be quantitated across multiple images and independent reps. Likewise for Fig 4 d,e.
- CX3CR1 staining was replaced with quantitative FACS analysis (now Fig. 2e, f), which appears to be more quantitative and specific. We also include quantitative analysis of the stainings for Nr4a1 (**Fig 2. g, h**).

**5)** Supp Fig 5 needs better images and quantitation from multiple images.
- Alternative images have been provided as requested (staining for CD11b, a myeloid marker and quantification included (**Supplementary Fig. 6a, b**).

**6)** Supp Fig 6 needs untreated or diluent treated control.
- Provided as requested: while the graphs showion exosome treatment are on log scale, the graphs for PEDF treatment include baseline (0) lebels (circled, now **Supplementary Fig. 7**)

**7)** It is not so clear that the macrophage depletion experiments are "all or nothing" in terms of mediating the effects of NM-Exo and the NK depletion experiments are "partial effect". That is, the differences observed with immune cell depletion look similar for macrophage depletion and NK depletion although the statistics say otherwise. Some revisiting of the statistical analysis, the n value, and interpretation of the data may be warranted. It's not that I think the authors are so off in their conclusions but it seems likely that with added data, the statistics might be similar.
- We agree with the reviewer and have moderated our statement ('the *in vivo* data suggests significant contribution of the NK cells in the anti-metastatic effect of Ex$^{NM}$; however, the *in vitro* data indicate NK-autonomous effects, which include tumor cell killing by fratricide and phagocytosis by macrophages', **pp. 8, 11**).

**9)** The actual n value that goes into statistics for all quantitations should be stated in the figure legends
- Provided as requested

**10)** To show conclusively that PEDF is responsible for the macrophage-promoting effects of NM-Exo, depletion of PEDF (e.g. by shRNA in the exosome-producing cells) from NM-Exo should be done and then the effect of equal numbers of PEDF-depleted Exo should be compared to control Exo from the same cell line. The neutralizing

antibody is not good enough, because the antibody may just prevent the exosomes or other proteins on the exosomes from interacting with cells.

- To address this issue, we performed additional experiments on macrophages using purified recombinant PEDF where it was sufficient to mimc the effects of ExoNM on macrophage differentiation (**p. 9, Fig. 5g**). Together with the use of antibodies, we feel in conclusively demonstrates the role of PEDF in macrophage-associated effects.

**11)** Fig 7, the statement that Peinado et al. found more exosomes in recurrent patients is not exactly true. To be precise, they reported finding no difference in exosome number in patients of any stage vs. normal, but did find more exosomal protein in Stage IV. I also don't know how much can be said about exosomal protein when the exosomes were isolated by ExoQuick (Fig 7a).

- We agree and have removed both the statement and the figure panel. Instead, we provide a more detailed characterization of the patient-derived exosomes (**Fig. 6a, Supplementary Fig. 10**). Also, please see calrification for isolation procedure, which included affinity opurification step following ExoQuick (**p. 14-15**).

**12)** The media used for collecting exosomes should be specified. "Exosome-free" media could mean many things.

- For exosome collection the cells were grown in defined media supplemented with serum depleted of exosomes purchased fom Life Technologies (**p. 14**).

**13)** At least some key experiments should be repeated using density gradient purified exosomes to show validation by an extra-pure vesicle population.

- When cell lines were used as a source, exosomes from cell lines were isolated by ultracentrifugation and not by ExoQuick, as is indicated in Methods secion. We apologize for the confusion. The quality of preparations was ascertained by TEM, dynamic light scattering and nanosight. Since we were performing multiple in vivo experiments, we were unable to produce preparative amounts of exosomes using gradient-based methods. However, ultracentrifugation is used in multiple papers by the leading grops in the field (Hoshino et al., Nature 2015. 527 (7578): 329-35. PMID: 26524530; Melo et al., Nature. 2015. 523(7559): 177-82. PMID: 26106858). One new experiment was done using density gradient (**Supplementary Fig. 8**) and shows PEDF localization to exosome fractions.

**14)** It would be good to have some non-exosome markers on the Western blots in Fig 1

- We have added GM130 and TBP as controls (not found in exosome preparations) (**Fig. 1b**).

**Minor Points:**

**15)** More referencing for role of immune cells in metastatic cell clearance, e.g. Malladi et al., Cell, 2016 for NK cells

- Provided as requested

**16).** Some reference for the isogenic C8161 cell lines should be given.

- Provided as requested.

**Reviewer #4:**

Understanding the role of exosomes in tumor progression, and in particular at the pre-metastatic niche, is most warranted. The potential novelty of this work relates to the anti-tumor effect of exosomes that are secreted by non-metastatic melanomas, by inducing an immune surveillance at the pre-metastatic niche. Further studies with important controls are required to substantiate the key conclusions offered by this study.

**Major:**

**Figure 1:** The effect could be due to an enhancement of metastasis induced exosM (as already published by Peinado et al, Nat Med 2012). Before the conclusions can be drawn, the authors should provide a control which consists of administering A375 cells after conditioning the mice with a vehicle. Along the same lines, does the conditioning with exoNM inhibit basal metastasis of A375 cells?

- This control was justly requested by all reviewers and provided for all key experiments. Basal metastasis is consistently inhibited by ExoNM (see response to the Reviewers 1 and 2 and **Figs. 1-4**).

**Supplementary figure 1:** What is the difference in exosome secretion in terms of quantity of vesicles? How many particles 10ug refers to? It has been published that exosomes from metastatic melanoma cells are enriched in proteins. Therefore, the authors should refer to a quantity of exosomes and not to a protein quantity.

- We did not observe major differences in exosome numbers/vume/ ug protein between metastatic and non-metastatic cell lines. However, there was consisgtent 2-fold difference between mouse and human cell lines. For in vivo experiments, we equalized exosome numbers ($2.62x10^8$/mouse, 10 µg and 20 µg of protein for mouse and human cells, respectively). Now indicated on **p. 14** and **Supplementary Fig. 1**, legend. Unfortunately, we had not enough human exosomes for nanotracking experiments.

**Supplementary Figure 4:** One expects to see a comparison of the biodistribution of exos NM and exosM in mice. Is this the case?

- The requested result is included (**Supplemetary Fig. 4**).

**Figure 3:** Staining of more specific markers of PMo than CX3CR1 should be performed in the lungs of mice pre-conditioned with ExoNM. Again, a control of mice treated with the vehicle should be included.

- We have added Nr4a1 staining (**Fig. 2g, h**), and FACS of alveolar myeloid cells for CX3CR1 (**Fig. 2e, f**), with similar results. Untreated controls were included as requested. We believe that these results demonstrate increased presence of the PMo in the lung using three independent methods.

**Figure 4e:** Phagocytosis of fluorescent-melanoma cells should be quantified.

- Quantification was provided per request of Reviewers 2 and 3 (**Fig. 3f**).

**Figure 6a:** To demonstrate that PEDF is enriched in exosomes, equal amounts of proteins of whole cell and exosome lysates should be analyzed by western blot in a panel of melanoma lines (metastatic or not) without manipulation of PEDF.

- Exosomal PEDF reflects its amount in cell lysates. There may be some mild enrichment, but this is not the point of the study. The requested figure has been included (**Supplementary Fig. 8a**).

**Supplementary figure 8:** Which treatment is represented by these images? Where are the other conditions (non-treated, exosNM and exosM treated)?

- To clarify our point, **Supplementary figure 9a** now includes three conditions (control, $Exo^{M}$, $Exo^{NM}$) and showns that apoptosis is restricted to melanoma cells. In **Supplementary Fig. 9b, c** all cells were treated with $Exo^{NM}$ to show that apoptosis was dependent on the presence of macrophages; a more detailed figure legend has been generated to better reflect the experimental design.

**Figure 7:** PEDF level in exosomes isolated from the patients would improve the message of this study.

- PEDF assessment in patients exosomes was conducted and shown in **Fig. 6d**.

What is the quantity of exosomes injected in each condition?

- See response to comment above

**Minor:**

**Sup1a and b:** Font too small, not readable

- The figure was replaced to provide better quality images

**Figure 2b:** The authors state ‘in the total myeloid population, Ly6Chi subpopulations were similar upon treatment with control ExoM or with ExoNM. However, only ExoNM caused a significant increase of the Ly6Clow population’. Cells are either Ly6C high or low. If there is a decrease in Ly6C low subpopulation percentage, the LyC high subpopulation should also be affected. This is what seen on the flow plots. This sentence should be changed.

- Changed as requested

‘In previous studies by our laboratory as well as other groups, it was established that pigment epithelium-derived factor (PEDF) renders melanoma cells non-metastatic. Importantly, PEDF is also expressed by most non-metastatic cell lines19’: Another reference is required.

- References added as requested

Reviewers' comments:

Reviewer #1 (Remarks to the Author):

The authors have added additional data substantially improving the manuscript. However, there is one key issue that is not addressed and few more minor issues which needs to be taken care.

1, Key issue: The exosomal PEDF data relies heavily on the antibody. This is weak as pointed out by all the reviewers. The authors have not addressed this major point other than using recombinant PEDF only to macrophages. The authors need to knockdown PEDF from these cells or from the sBCL2 cells to attribute this to PEDF. As the metastatic cells are established by overexpression (unclear in first version), this data needs to be validated on cells with endogenous levels of PEDF (e.g., sBCL2). In other words, can the authors show that loss of PEDF in sBCL2 exosomes induce more metastasis compared to wild type exosomes?
2, Key issue: The authors show that NM exosomes reduce metastasis through PEDF. The authors also show that PEDF can induce macrophage differentiation similar to NM exosomes. One critical data that is missing from this article is whether recombinant PEDF by itself can reduces metastasis? Can the authors provide this data? This direction have therapeutic implications to this study. May be MSC exosomes with PEDF or recombinant PEDF can block metastasis in melanoma patients. The authors can also add these in discussion to increase the impact of the story.
3, Fig 5b – after trypsin digestion, is there a shift in the molecular weight of PEDF? If not, the figure is not aligned well. If there is shift, please mention in the manuscript/legend. Also, how much protein was loaded?
4, Fig 6a – the magnified rectangle is not the same. Left side EVs are not selected in the inset within.
5, Except one cell line (sBCL2), all the others in Fig 5a have overexpressed PEDF.

Reviewer #2 (Remarks to the Author):

A number of things have been fixed (the major stuff) but a few minor remaining issues need to be resolved.

1. The statistical comparisons between between experiments in which cells or mice have been treated with Exo-NM or Exo-M or UnTx seem to only make the comparisons between Exo-NM and the other two conditions. Exo-M should also be compared statistically with UnTx to see replication of previous Lyden studies and any other effects of Exo-M (or not).
2. Fig 1 legend error: calls B16F10 melanoma cells human whereas they are mice.
3. Fig 3f, are the differences not statistically significant? Look like they should be but is not indicated
4. Double check references to Fig numbers, not all are correct.

Reviewer #4 (Remarks to the Author):

authors have largely addressed the reviewer comments, and the manuscript has been therefore improved.

Response to reviewers' comments:

Enclosed, please find the revised version of the manuscript NCOMMS-16-21838A-Z entitled 'Pre-metastatic cancer exosomes induce immune surveillance by patrolling monocytes at the metastatic niche'. We truly appreciate the reviewers' positive outlook and attemted to addressed their additional comments to the best of our ability. We believe that these changes have further improved the manuscript and hope it to be now acceptable for publication in Nature Communications. Below we delineate the changes made to accommodate specific concerns.

Reviewer 1.

1. Key issue: As pointed out by all the reviewers the exosomal PEDF data relies heavily on the antibody. The authors have not addressed this major point other than using recombinant PEDF to treat macrophages. As the metastatic cells are established by overexpression, this data needs to be validated on cells with endogenous levels of PEDF Can the authors show that loss of PEDF in exosomes results in more metastasis compared to wild type exosomes?
   - We have used poorly aggressive, non-metastatic C81-81 PA cells, which endogenously express significant levels of PEDF; PEDF expression was silenced using shRNAmiR as was published previously (Orgaz and Ladhani, Oncogene Oncogene. 2009 Nov 26;28(47):4147-61) and the lack of PEDF in exosomes verified by Western blot (Supplementary Fig. 13). We now demonstrate that upon PEDF knockdown the ability of C81-61 exosomes to suppress metastasis is significantly diminished (Fig. 5d and p.9 of the manuscript).
2. Key issue: The authors show that NM exosomes reduce metastasis through PEDF and that PEDF can induce macrophage differentiation similar to NM exosomes. Can the authors provide data that show recombinant PEDF to reduce metastasisThis direction add therapeutic implications to this study. The authors can also add these consideration in discussion to increase the impact of the story.
   - Study by pothers shows the anti-metastatic effect of liposomal PEDF against melanoma metastasis to the lung (Shi et al, J Transl Med. 2013 Apr 3;11:86. doi: 10.1186/1479-5876-11-86). We have used PEDF active peptide mimetic that reproduces PEDF tumo-suppressive and anti-metastatic activity (Mirochnik et al, Clin Cancer Res. 2009 Mar 1;15(5):1655-63; Ladhani and Orgaz Neoplasia. 2011 Jul;13(7):633-42.) The results demonstrating the inhibition of B16F10 melanoma metastasis by PEDF peptide are now presented as Supplementary Figure 14 and p. 9 of the main manuscript
3. Fig 5b – after trypsin digestion, there a shift in molecular weight of PEDF after Trypsin digestion? If there is shift, please mention in the manuscript/legend. Also, how much protein was loaded?
   - A statement to that effect is now included on p. 9. Protein loading is included in figure legend and in Supplementary data.
4. The magnified rectangle in Fig. 6a is not accurate
   - We thank the reviewer for pointing this out and made an appropriate adjustment in the figure.
5. Except one cell line all melanoma cell lines overexpress PEDF.

- This is not quite the case: C81-61 PA is naturally expressing high levels of PEDF and is non-metastatic. It has been used for PEDF knockdown experiment. We attempted to clarify the statement in this section (p. 8 and appropriate figure legend).

Reviewer 2.

1. The statistical comparisons between between experiments in which cells or mice have been treated with Exo-NM or Exo-M or UnTx seem to only make the comparisons between Exo-NM and the other two conditions. Exo-M should also be compared statistically with UnTx to see replication of previous Lyden studies and any other effects of Exo-M (or not).
    - The comparison has been added to all relevant figures. Our results corroborate the results by the Lyden group and show a significant increase in ExM treated group over control (Untx).
2. Legend error in figure 1 (B16 melanoma referred to as human cell line)
    - We thank the reviewer for pointing this out an made the appropriate correction.
3. Fig. 3f does not have statistical analysis included
    - Provided as requested
4. Double-check references to figure numbers:
    - Done as requested

We thank the Reviewer 3 for his previous suggestions (none was made to the revised version of the manuscript).

REVIEWERS' COMMENTS:

Reviewer #1 (Remarks to the Author):

The authors addressed the issues raised.

Reviewer #2 (Remarks to the Author):

Statistical comparisons between Exo-M and UnTx are still missing for a few figures: Figs 2f, 4d, Supp Fig 7.

**Reviewers' comments:**

Reviewer #1:

The authors addressed the issues raised.

Reviewer #2:

Statistical comparisons between Exo-M and UnTx are still missing for a few figures: Figs 2f, 4d, Supp Fig 7.

**Response to reviewers' comments:**

We are sincerely grateful to the reviewers for their patience and positive view of our work. There were no additional comments from Reviewer 1. In response to the Reviewer 2 comments, we have added statistical comparisons between Exo-M and UnTx conditions to the figure panels 2f, 4d, and Supplemental Fig. 7.
